# Mechanical characterization and transversely isotropic finite element modelling of sugarcane stalk

**DOI:** 10.3389/fpls.2026.1938979

**Published:** 2026-09-16

**Authors:** Zhenhui Zheng, Qingyu Guo, Yingcong Chen, Dongjie Du, Shuo Wang, Weihua Huang, Lijiao Wei, Ming Li

**Affiliations:** 1 Agricultural Machinery Research Institute, Chinese Academy of Tropical Agricultural Sciences, Guangdong, China; 2 College of Engineering, South China Agricultural University, Guangdong, China; 3 Key Laboratory of Agricultural Equipment for Tropical Crops, Ministry of Agriculture and Rural Affairs, Guangdong, China

**Keywords:** elastic modulus, finite element model, mechanical properties, sugarcane stalk, transverse isotropy

## Abstract

**Background:**

Sugarcane stalks are subjected to compression, clamping, bending, feeding, and conveying during mechanised harvesting, but their mechanical behaviour is commonly characterised under individual loading conditions, limiting the representation of directional and longitudinal variability in numerical models.

**Methods:**

This study establishes an integrated experimental and modelling framework to characterise these variations and incorporate them into a transversely isotropic finite element model. Mature Guitang 21 stalks were tested at basal and middle internodes using axial compression, radial compression, and three-point bending.

**Results:**

The basal internodes exhibited an axial elastic modulus of 96.95 MPa, an equivalent radial deformation parameter of 17.66 MPa, and an equivalent bending response parameter of 174.10 MPa, compared with 65.90 MPa, 14.47 MPa, and 140.39 MPa, respectively, for the middle internodes.

**Conclusions:**

These results reveal two consistent mechanical features: substantially greater resistance along the stalk axis than in the radial direction, and systematically greater mechanical resistance in the basal region than in the middle region. The quantified parameters and the validated model provide a basis for refining the design of clamping, feeding, and cutting components in mechanised harvesting machinery.

## 1 Introduction

Sugarcane is an important sugar and energy crop that is widely used in sugar production, biofuel production, and the paper industry (Wu et al., 2024; Que et al., 2024). China is one of the major sugarcane-producing countries in the world, with both cultivation area and production ranking among the highest globally. Among its production regions, Guangxi represents the largest sugarcane-producing area in China (Lu et al., 2019; Martins et al., 2023; Mo et al., 2024; Xu et al., 2023).With the continuous increase in rural labour costs and the growing demand for large-scale sugarcane production, the mechanization of the entire production process has become an important approach to improving industrial competitiveness. During mechanized harvesting, sugarcane stalks undergo a series of operations, including cutting, clamping, feeding, conveying, and billet cutting. The mechanical response of the stalk during these processes directly affects cutting energy consumption, conveying stability, and the level of stalk damage. Studies have shown that relatively high rates of top-breaking and mechanical damage still occur during mechanized harvesting, which adversely affects seed cane quality and subsequent sprouting performance (Qian et al., 2024; Lai et al., 2026). Due to the internal structure of sugarcane stalks, which consists of epidermal tissue, vascular bundles, and pith, they exhibit clear heterogeneity and anisotropic characteristics, resulting in complex mechanical behaviour. Accurate determination of the mechanical properties of sugarcane stalks and the development of reliable numerical models are essential for improving harvesting equipment design, reducing damage rates, and enhancing the quality of mechanized harvesting operations.

To date, extensive research has been conducted on the mechanical properties of sugarcane stalks and other similar plant stem materials (Ma et al., 2024; Shen et al., 2023; Huo et al., 2024; Chen et al., 2016; Liu et al., 2022). Existing studies have primarily focused on the determination of strength and elastic parameters under compression, bending, tensile, and shear loading conditions (Xie et al., 2018; Omprabha et al., 2025). Some studies have employed axial compression and three-point bending tests to characterize the compressive and bending behaviours of sugarcane stalks. The results indicate that their mechanical properties vary significantly with internode position and moisture content (Li et al., 2023; Abdelhadi et al., 2023; Al-Zube et al., 2018).In addition, studies on natural fibre-reinforced biological materials such as maize stalks, bamboo, and banana peduncles have shown that their elastic modulus and failure behaviour exhibit clear directional dependence and structural heterogeneity (Chen et al., 2016; Liu et al., 2022; Liese and Köhl, 2015; Greco et al., 2024; Xie et al., 2018; Thompson et al., 2019). With the development of numerical simulation techniques, the finite element method has been widely applied to the analysis of mechanical behaviour in agricultural biomaterials. Related studies have developed finite element models for materials such as apples, tomatoes, maize stalks, and banana peduncles to predict deformation and damage processes under loading, demonstrating the applicability of the finite element method in biomechanical studies of agricultural materials (Li et al., 2025; Sharma et al., 2015; Dintwa et al., 2008; Li and Wang, 2016). However, the systematic characterisation of the mechanical parameters of sugarcane stalks remains limited. In particular, there is still a lack of systematic studies on the determination of direction-dependent mechanical parameters, the development of transversely isotropic constitutive models, and the experimental validation of such models.

The vascular bundles and fibrous tissues within sugarcane stalks are directionally distributed along the longitudinal axis, resulting in typical transversely isotropic characteristics. Although previous studies have reported the mechanical properties of sugarcane stalks under compression, shear, and bending loading conditions, most of them have focused on parameter determination under a single loading mode. Systematic investigations into the relationships among axial, radial, and bending mechanical properties remain limited (Liu et al., 2024; Ma et al., 2024).At the same time, existing finite element models are often based on homogeneous or isotropic material assumptions, which are insufficient to capture the anisotropic behaviour arising from the directional distribution of fibrous tissues within sugarcane stalks (Liese and Köhl, 2015; Li et al., 2025). Studies on transversely isotropic finite element models established using experimentally measured mechanical parameters and evaluated through experiments remain relatively limited (Wenzel et al., 1989; Lai et al., 2026).In addition, many existing finite element studies have adopted simplified isotropic material properties, which cannot adequately represent the fibre-reinforced structural characteristics of sugarcane stalks. In particular, there is a lack of a complete methodological framework integrating mechanical parameter characterisation, transversely isotropic constitutive modelling, and experimental assessment of model performance under representative loading conditions. This limitation restricts the agreement with experimental measurements of finite element models in the design of mechanised sugarcane harvesting equipment (Qiu et al., 2021; Gangwar et al., 2021; Zhou et al., 2024; Niklas, 2000).To address these gaps, mature sugarcane stalks of the cultivar Guitang 21 were selected in this study. Axial compression, radial compression, and three-point bending tests were conducted on basal and middle internodes to determine the axial elastic modulus (E_z_), equivalent radial deformation parameter (E_r_), and equivalent bending response parameter (E_b_). Correlation analysis and significance testing were further performed to investigate directional mechanical behaviour and longitudinal mechanical gradients. Based on the experimentally informed mechanical parameters, a transversely isotropic finite element model was established and its bending response was evaluated through comparison with the three-point bending experiments. The results provide a theoretical basis for the mechanical analysis, numerical simulation, and structural optimisation of sugarcane harvesting equipment. The overall workflow is shown in **Figure 1**.

**Figure 1:**
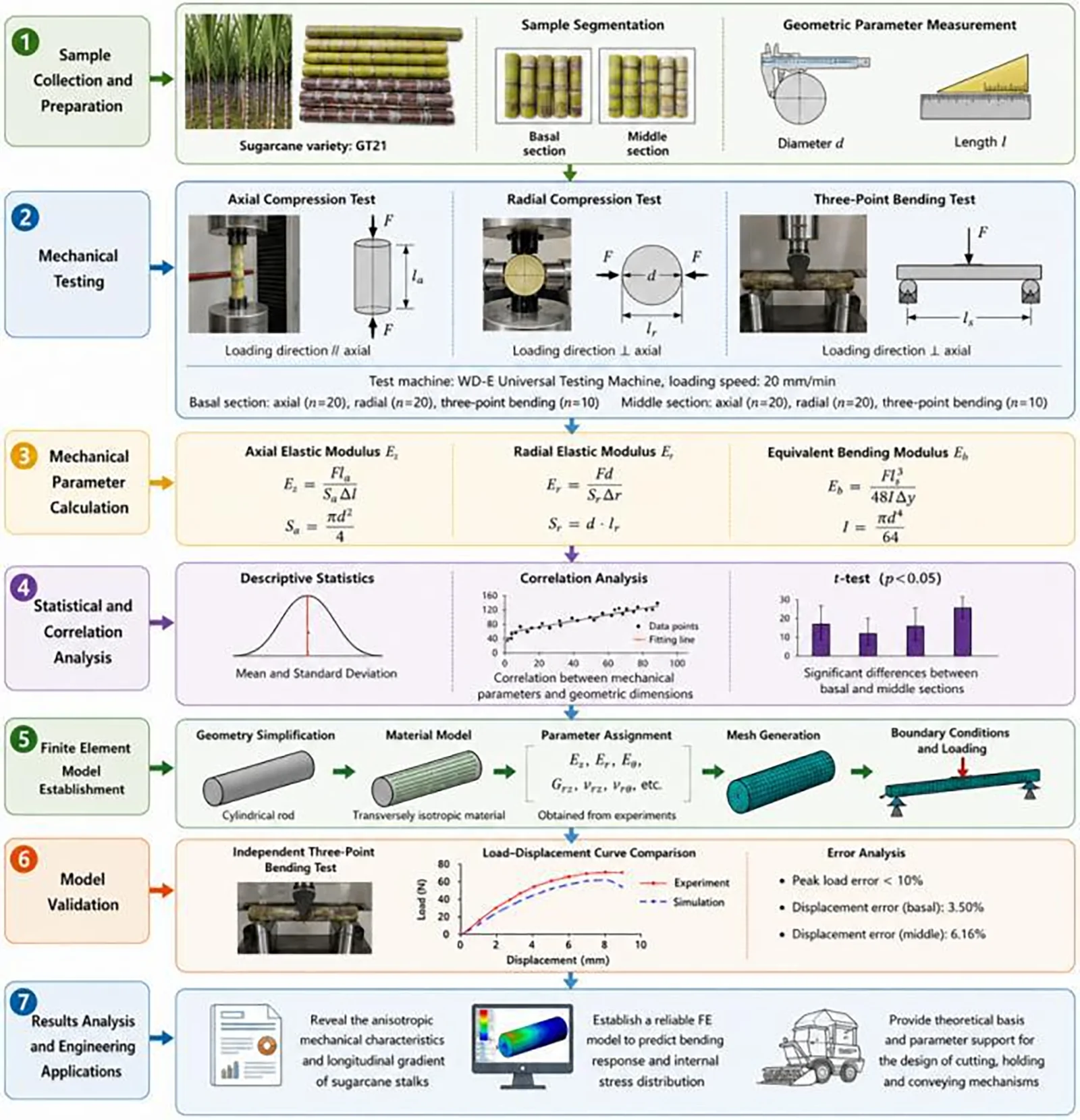
Overall workflow diagram, where E_z_ denotes the axial elastic modulus, E_r_ denotes the equivalent radial deformation parameter, and E_b_ denotes the equivalent bending response parameter.

## 2 Materials and methods

### 2.1 Sample preparation and morphological characteristics

The sugarcane stalk samples used in this study were collected from a plantation in Zhanjiang, Guangdong Province, China, and the cultivar was Guitang 21. The samples were harvested at the mature stage, when the internodes were fully developed, the epidermis appeared yellow-green, and no obvious signs of disease, pest infestation, or mechanical damage were observed. To ensure the stability of mechanical properties, all tests were completed within 48 hours of harvest. During transportation to the laboratory, the stalks were immediately placed in insulated containers with ice packs to minimize moisture loss and prevent tissue senescence. Prior to testing, the specimens were sealed in polyethylene bags and stored at room temperature (approximately 26°C) for short-term preservation to minimize the effect of moisture loss on mechanical behaviour. A total of 40 mature sugarcane stalks were collected and considered as independent biological replicates, with each stalk treated as one experimental unit. Two longitudinal positions (basal and middle sections) were selected from each stalk; thus, each individual stalk contributed both basal and middle section samples. For axial and radial compression tests, specimens were obtained from the corresponding sections of each stalk. For three-point bending tests, some stalks provided two specimens from the same sampling position to ensure measurement repeatability. These multiple specimens from the same stalk were treated as repeated observations within the same biological replicate rather than as independent biological samples. For statistical comparisons between basal and middle sections, measurements were matched according to stalk identity. When multiple specimens were obtained from the same stalk and sampling position, these repeated observations were first summarized at the stalk level and were not counted as additional independent biological replicates. After removing surface impurities, stalks with uniform growth, intact morphology, and no visible defects were selected for testing. Based on the longitudinal structural characteristics of sugarcane stalks, the specimens were divided into basal and middle regions. The basal region was defined as the first to third internodes near the root, while the middle region comprised the fourth to eighth internodes, as shown in **Figure 2**. The stalks were then cut into specimens with lengths ranging from 60 to 100 mm.

**Figure 2:**
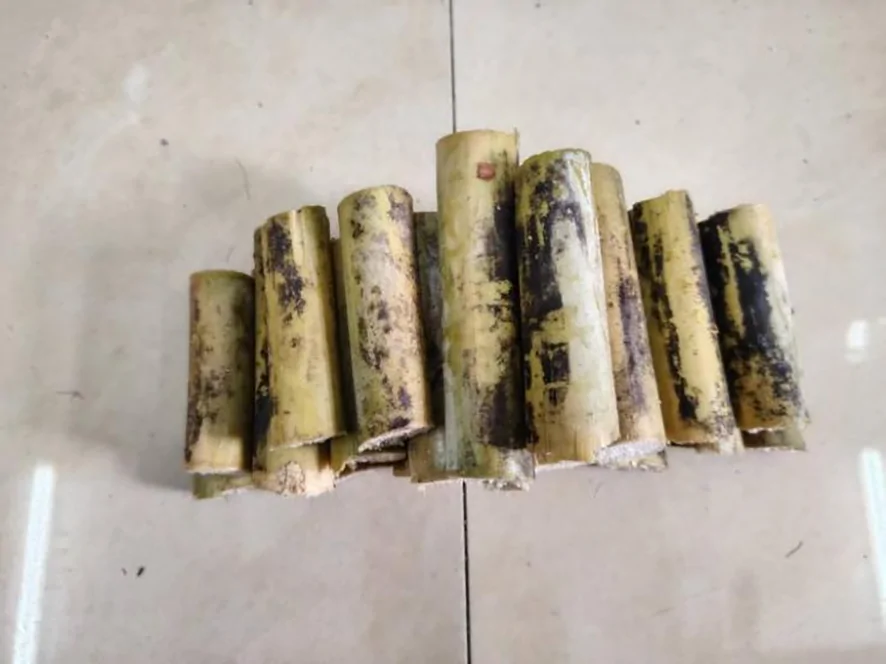
Experimental sugarcane stalk samples.

To investigate the mechanical properties under different loading conditions, a total of 100 specimens were prepared, including 40 sets for axial compression tests (20 from the basal region and 20 from the middle region), 40 sets for radial compression tests (20 from the basal region and 20 from the middle region), and 20 sets for three-point bending tests (10 from the basal region and 10 from the middle region). By comparing the mechanical responses of specimens from different internodal positions, the longitudinal gradient in mechanical properties of sugarcane stalks and the differences between regions were analysed.

The moisture content of the specimens was not experimentally measured in this study. The preservation procedures described above were intended only to minimise moisture loss between harvesting and mechanical testing and should not be interpreted as control or measurement of specimen moisture content. Accordingly, moisture content was not included as an experimentally measured variable in the subsequent analysis. According to previous studies on fresh sugarcane stalks harvested under similar conditions, the moisture content is generally reported to be approximately 70%–78% (Speck and Burgert, 2011; Niklas, 2013). Therefore, this reported range was used only to describe the typical moisture condition of fresh sugarcane stalks during mechanical testing.

During specimen preparation, to reduce the influence of local structural discontinuities on the experimental results, relatively straight stalk segments were selected as far as possible, and the node regions were avoided to minimise the effect of structural heterogeneity associated with internodes on the mechanical response. The geometric dimensions of each specimen, including the length (l) and the cross-sectional diameter (d), were measured using a vernier calliper. Considering that sugarcane stalks are approximately slender cylindrical structures and that variations in cross-sectional dimensions were relatively limited, the specimens were simplified as circular cross-sectional rods for mechanical parameter calculations and finite element modelling. This simplification preserves the primary macroscopic geometric features of sugarcane stalks while standardising the loading area, second moment of area, and geometric representation. As a result, consistency and comparability between mechanical parameter estimation and finite element analysis are improved. These geometric parameters were subsequently used in mechanical property calculations, dimensional correlation analysis, and finite element model development.

The main object of study in mechanised harvesting and conveying of sugarcane is the whole sugarcane stalk. Its structure primarily consists of the root, stalk, top (shoot), nodes, and internodes, as shown in **Figure 3**. Among these components, the root and stalk bear the main mechanical loads, while the structural differences between nodes and internodes are key factors leading to the anisotropic behaviour and local heterogeneity of the mechanical properties of sugarcane stalks.

**Figure 3:**
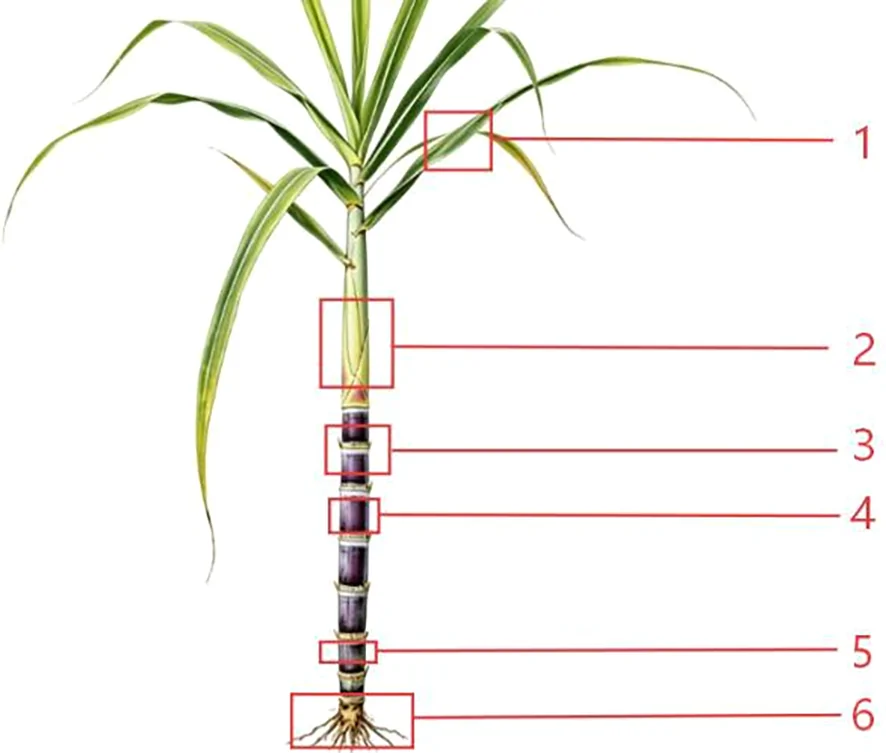
Schematic diagram of sugarcane structure.

No specimens from the axial compression, radial compression, or three-point bending tests were excluded from the final analyses; all specimens that completed the prescribed tests were retained.

### 2.2 Determination of mechanical parameters

#### 2.2.1 Experimental preparation stage

All mechanical tests were conducted under consistent ambient conditions (temperature:26°C, relative humidity:82%) to minimize environmental influences.

The internal structure of sugarcane stalks consists of epidermal tissue, vascular bundles, and pith tissue. The fibrous components are directionally aligned along the longitudinal axis, exhibiting typical transversely isotropic characteristics. During mechanised harvesting, sugarcane stalks are subjected to complex loading conditions, including longitudinal compression, transverse clamping, and bending deformation. The mechanical responses under different loading directions differ significantly. To systematically characterise the macroscopic mechanical behaviour of sugarcane stalks, axial compression, radial compression, and three-point bending tests were conducted in this study. These tests were used to characterise the mechanical responses under longitudinal compression, transverse compression, and bending-dominated conditions, respectively, using the axial elastic modulus (E_z_), equivalent radial deformation parameter (E_r_), and equivalent bending response parameter (E_b_). Unlike E_z_, E_r_ and E_b_ are engineering response parameters derived under specific loading configurations and should not be interpreted as intrinsic material moduli. Considering that sugarcane stalks are natural bio-composite materials with a complex internal structure and pronounced local heterogeneity, this study focuses on the macroscopic mechanical response of the stalk as a whole, rather than the local mechanical behaviour of individual tissues at the microscale. Therefore, all measured mechanical parameters are treated as macroscopic equivalent parameters and are used for the subsequent development of a transversely isotropic finite element model and force analysis under typical loading conditions. By comparing mechanical parameters in different directions, the direction-dependent behaviour and longitudinal mechanical gradient of sugarcane stalks can be further characterised. This provides a parameter basis for the optimisation of cutting, clamping, and conveying components in mechanised sugarcane harvesting equipment.

All mechanical tests were conducted using a WD-E precision computer-controlled electronic universal testing machine (as shown in **Figure 4**). The equipment was manufactured by Guangzhou Guangcai Experimental Instrument Co., Ltd., with a maximum load capacity of 20000N. The measurement accuracy for both force and displacement was ±1%, and the loading rate was set uniformly at 20 mm/min. The displacement data used for strain calculation were obtained from the crosshead displacement of the testing machine. Although an extensometer was not used due to the small specimen size and irregular biological surface characteristics, the same displacement measurement method was applied consistently for all specimens to ensure comparability among samples. For axial compression and radial compression tests, 40 parallel specimens were prepared for each test type (20 from the middle region and 20 from the basal region). For the three-point bending test, 20 specimens were used in total, including 10 basal and 10 middle specimens, in order to reduce the influence of individual variability on the experimental results.

**Figure 4:**
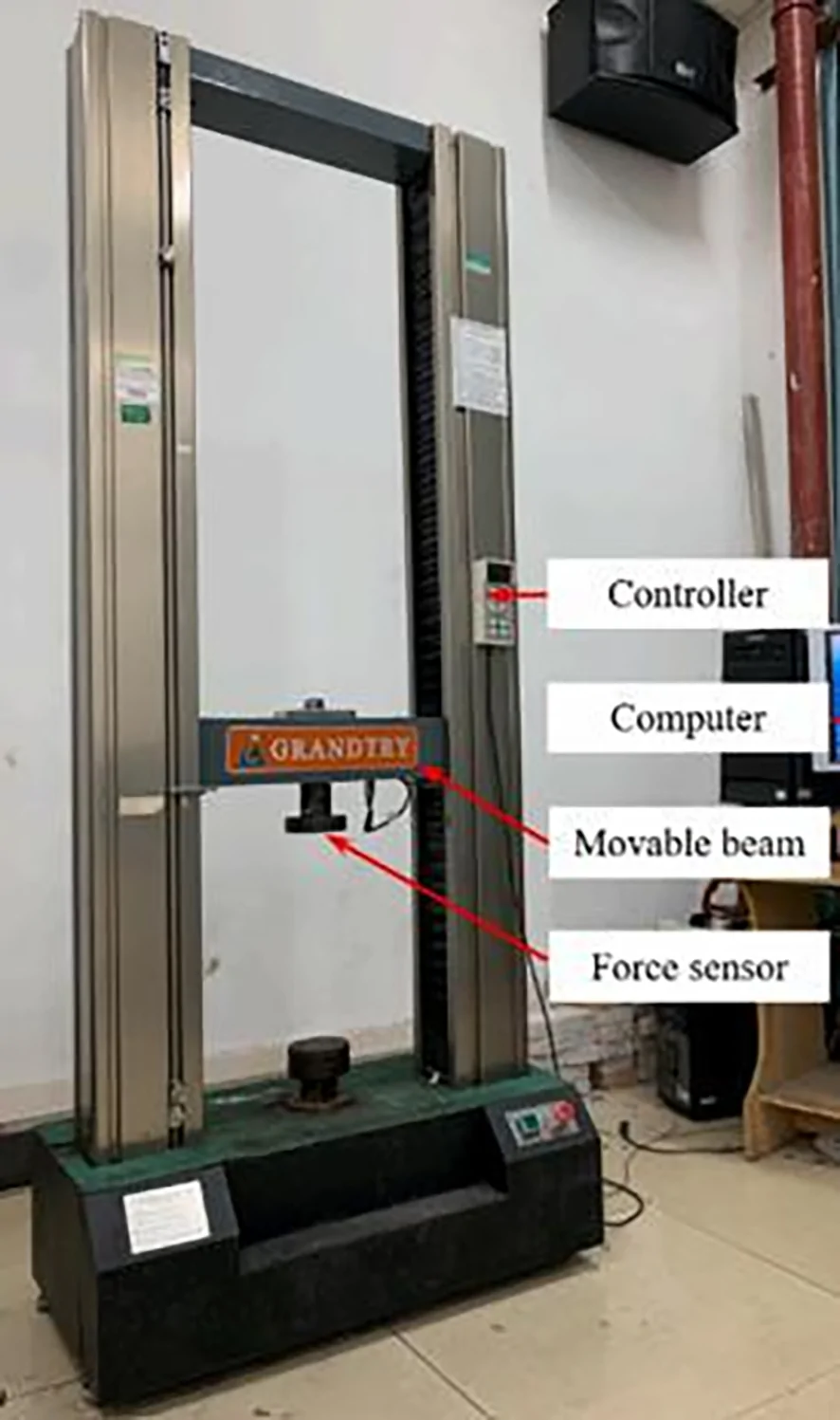
WD-E precision computer-controlled electronic universal testing machine.

#### 2.2.2 Axial compression test

For the determination of the axial elastic modulus, specimens from the middle and basal regions were placed vertically on the supporting platform, with the loading direction aligned with the longitudinal axis of the sugarcane stalk (as shown in **Figure 5**), to compare the axial mechanical responses at different internodal positions. To minimise the effects of end-surface irregularities and eccentric loading on the results, both ends of all specimens were carefully flattened, and the loading direction was ensured to be aligned with the specimen axis. Before formal compression loading, a small preload was applied to ensure complete contact between the specimen end surfaces and the loading platen. The preload-induced displacement was considered as seating deformation and was excluded from modulus calculation. During testing, specimen alignment was visually checked to minimise eccentric loading. Specimens showing obvious local crushing, unstable deformation, or abnormal contact conditions were predefined for exclusion from subsequent analysis. However, no specimens met these exclusion criteria, and all prepared specimens were retained in the final analysis.

**Figure 5:**
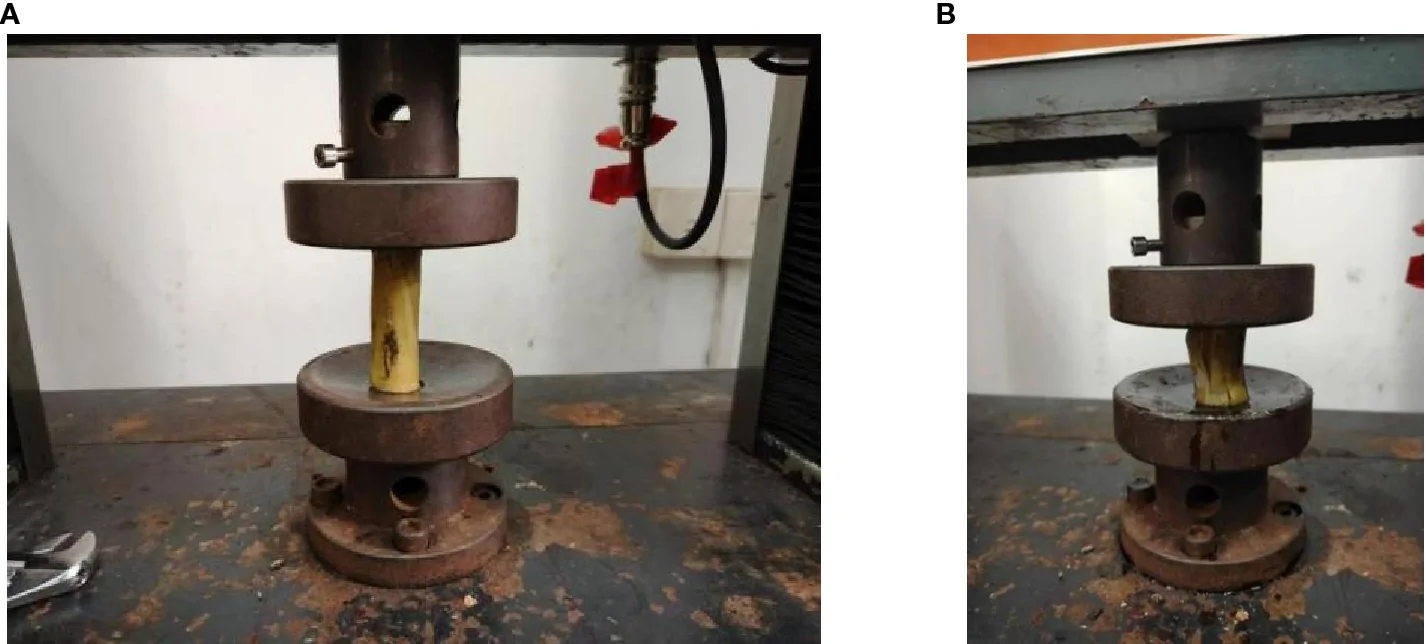
Axial compression test of sugarcane stalks: **(A)** basal specimens; **(B)** middle specimens.

By recording the load (F) and axial displacement (Δl) during the loading process, and combining the initial specimen length (l_a_) and cross-sectional area (S_a_), the axial stress and axial strain were calculated, from which the axial elastic modulus (E_z_) was obtained. The calculation principle is as follows, and the axial elastic modulus is calculated using Equation (1):

(1) $$E_{z}=\frac{Fla}{S_{a}\Delta l}$$

In this study, the axial elastic modulus was determined from the slope of the linear elastic region of the stress–strain curve. Before regression analysis, the initial seating region caused by the contact adjustment between the specimen and loading platen was excluded. The modulus was calculated by linear fitting within the selected elastic deformation interval rather than using a single secant or tangent value. The coefficient of determination (*R²*) of the linear regression was calculated to evaluate the reliability of the modulus estimation.

where E_z_ is the axial elastic modulus, F is the applied load, Sa is the axial loaded cross-sectional area, l_a_ is the initial axial length of the specimen, and Δl is the axial displacement. Since the cross-section of sugarcane stalks is approximately circular, the cross-sectional area can be expressed as Equation (2):

(2) $$S_{a}=1/4\pi d^{2}$$

where d is the diameter of the sugarcane stalk cross-section. This parameter is used to characterise the ability of the sugarcane stalk to resist compressive deformation along the longitudinal direction.

#### 2.2.3 Radial compression test

For the determination of the equivalent radial deformation parameter, specimens from the middle and basal regions were positioned for radial compression, with the loading direction perpendicular to the longitudinal axis of the sugarcane stalk (as shown in **Figure 6**), to compare the radial mechanical responses at different internodal positions.

**Figure 6:**
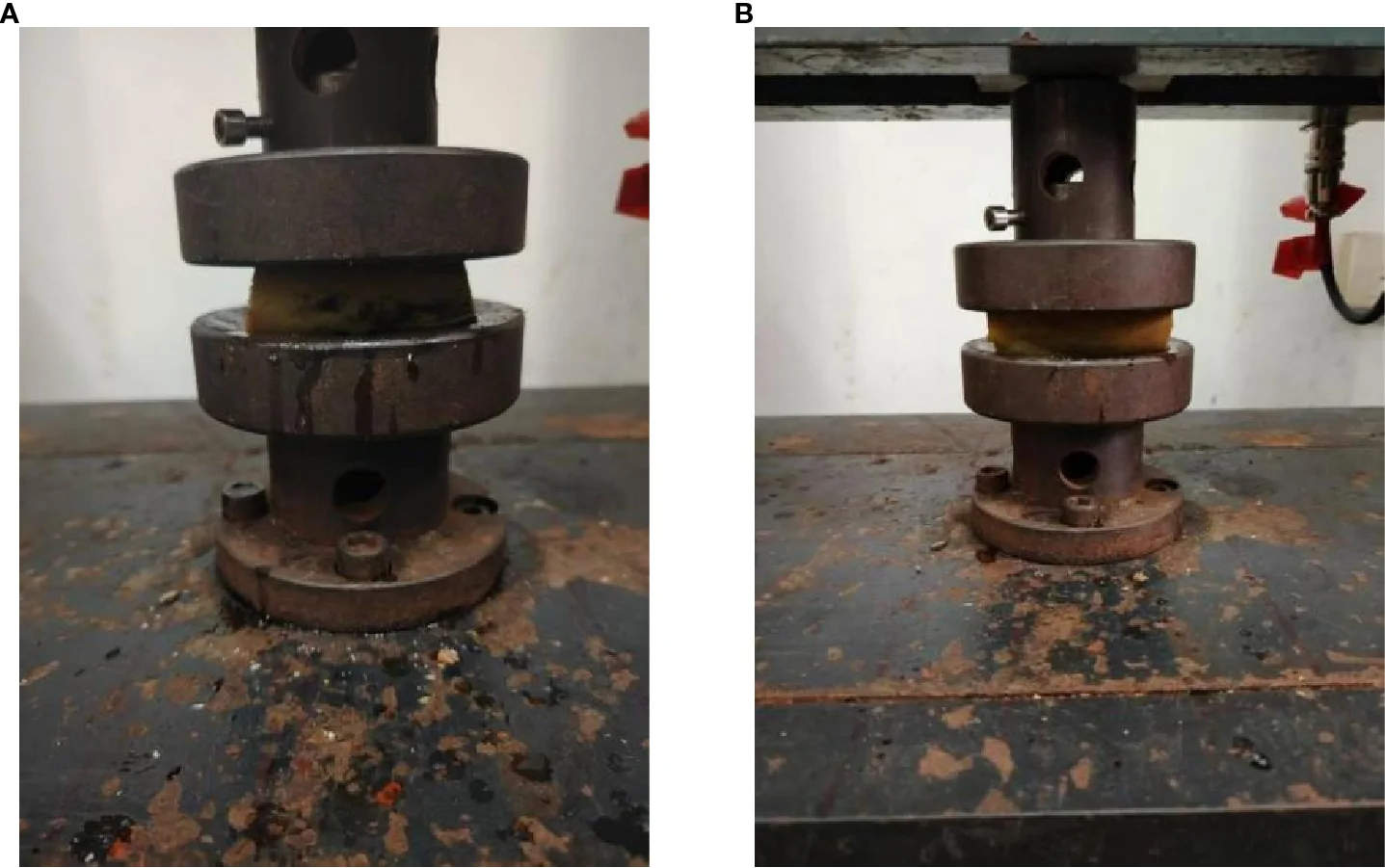
Radial compression test of sugarcane stalks.

During the loading process, the load (*F*) and radial displacement (*Δl_r_)* were recorded. The equivalent radial deformation parameter (E_r_) was calculated from the load–displacement response under radial compression. Because sugarcane stalks are heterogeneous biological structures with irregular internal tissues, Er represents a normalized measure of radial deformation resistance under the specific diametral compression configuration rather than an intrinsic transverse Young’s modulus. The calculation principle is as follows, and the equivalent radial deformation parameter is calculated using Equation (3):

(3) $$E_{r}=\frac{Fd}{S_{r}\Delta l_{r}}$$

Here, Er denotes the equivalent radial deformation parameter obtained from diametral compression testing. Due to the cancellation of diameter terms during derivation, this parameter should be interpreted as a normalized radial deformation resistance rather than an intrinsic transverse elastic constant. *S_r_* is the projected area under radial compression, and *Δl_r_* is the radial deformation. For the specimens used in this study, the projected area under radial compression can be expressed as Equation (4):

(4) $$S_{r}=dlr$$

where *l_r_* is the specimen length. This parameter primarily reflects the ability of the transverse tissues of sugarcane stalks to resist local collapse and lateral deformation under compressive loading.

#### 2.2.4 Three-point bending test

To characterise the overall deformation resistance of sugarcane stalks under bending-dominated loading conditions, a three-point bending test was conducted to determine the equivalent bending response parameter (E_b_), as shown in **Figure 7**. E_b_ was derived from the load–displacement response using the classical beam-bending expression as an engineering approximation. Because the measured response may include contributions from flexural deformation, transverse shear, local contact deformation, and specimen geometry, E_b_ is treated as a configuration-dependent engineering response parameter rather than an intrinsic elastic modulus.

**Figure 7:**
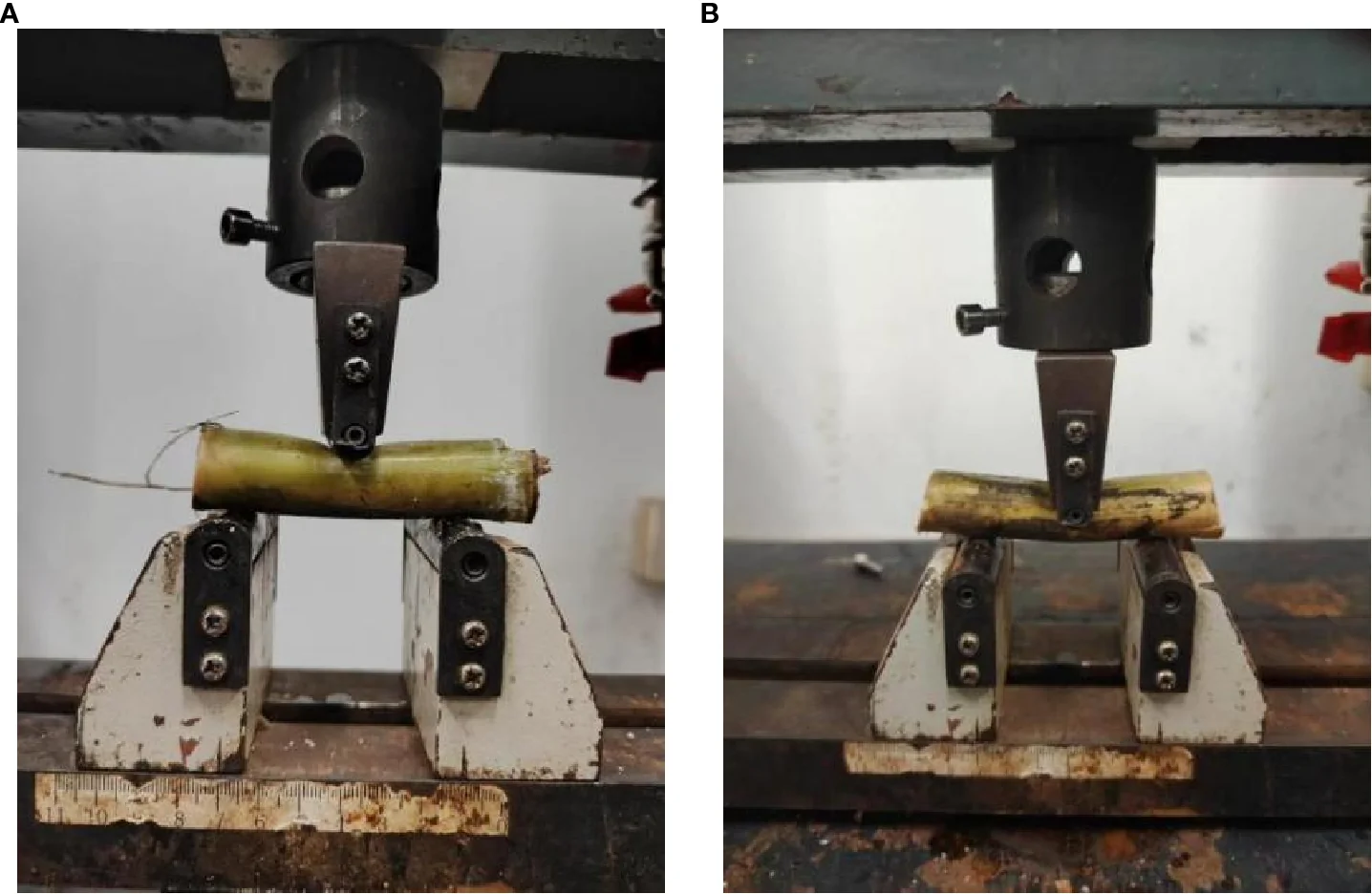
Three-point bending test.

In the experiment, specimens from the middle and basal regions were placed between two supporting supports, with a span length (*l_s_*) of 60 mm. The loading indenter was positioned at the mid-span and applied a downward load. The load (*F*) and mid-span displacement (*δ_s_*) during bending were recorded. Based on beam bending theory and the geometric parameters of the cross-section, the equivalent bending response parameter (E_b_) can be estimated. Due to the natural variability of sugarcane stalk geometry, the specimen span-to-depth ratio ranged from approximately 2.3 to 3.0 in this study. At these relatively small span-to-depth ratios, the assumptions of an ideal slender beam are not fully satisfied, and the measured mid-span displacement may contain contributions from transverse shear deformation and local contact deformation in addition to flexural deformation. Therefore, the classical beam bending equation was retained only as an engineering approximation to normalise the load–displacement response and obtain a comparable equivalent bending parameter under the present three-point bending configuration. The resulting equivalent bending response parameter should not be interpreted as an intrinsic Young’s modulus. The expression is given as Equation (5):

(5) $$E_{b}=\frac{Fl_{s}^{3}}{48{\delta s}_{I}}$$

where l_s_ is the support span, I is the second moment of area of the specimen cross-section, and δ_s_ is the mid-span displacement. For specimens with an approximately circular cross-section, the second moment of area can be approximated using the expression for a circular section, as shown in Equation (6):

(6) $$I=\pi d^{4}/64$$

In this calculation, transverse shear deformation, local contact deformation, and other non-ideal beam effects were not measured separately. Consequently, their individual contributions to the measured mid-span displacement and the calculated E_b_ could not be quantified from the available experimental data. This represents a limitation of the present bending analysis. Therefore, E_b_ should be interpreted as a configuration-dependent equivalent deformation parameter that incorporates flexural deformation together with possible shear, contact, and geometric effects. It should be noted that the circular cross-section assumption used in the calculation of the bending response parameter represents a macroscopic equivalent approximation rather than a description of the actual internal structure of sugarcane stalks. Although sugarcane stalks contain heterogeneous tissues, including rind, vascular bundles, and pith, the present calculation treats these components as an integrated structural entity. Therefore, the bending response parameter obtained from the three-point bending test is defined as an equivalent structural bending response parameter, which reflects the combined effects of material anisotropy, cross-sectional geometry, and internal tissue heterogeneity.

After the tests were completed, the axial elastic modulus, equivalent radial deformation parameter, and equivalent bending response parameter of each specimen were calculated using the above equations. The mean value, standard deviation, standard error, and coefficient of variation were further computed, and the relationships between the mechanical properties and the geometric dimensions of the sugarcane stalks were analysed. Considering that the specimen dimensions varied naturally among sugarcane stalk samples, the influence of specimen geometry was further evaluated. The aspect ratio (length/diameter) was calculated for each specimen, and linear regression analysis was conducted between aspect ratio and axial elastic modulus to determine whether geometric constraints significantly affected the measured mechanical parameters.

### 2.3 Finite element modelling and experimental comparison

#### 2.3.1 Basic assumptions for modelling

To further analyse the overall mechanical response of sugarcane stalks under mechanised operation conditions and to evaluate the applicability of experimentally obtained mechanical parameters in numerical simulations, a finite element model of the sugarcane stalk was developed based on the measured material properties. Considering that real sugarcane stalks exhibit complex structural features, including internode undulations, local tapering, non-uniform distribution of vascular bundles, and surface imperfections, while the present study focuses on their macroscopic mechanical behaviour, appropriate engineering simplifications were introduced in the modelling process.

Specifically, the sugarcane stalk was simplified as an equivalent cylindrical continuum with a circular cross-section, rather than a physically homogeneous equivalent cylindrical continuum. The heterogeneous tissues, including rind, vascular bundles, and pith, were not explicitly modelled at the microscopic scale; instead, their overall mechanical contributions were represented through direction-dependent equivalent elastic parameters. The longitudinal direction was defined as the principal material direction, while the radial direction was defined as the transverse direction. The model did not distinguish between epidermal tissue, vascular bundles, and pith at the microstructural level, and these were instead treated as an equivalent homogeneous continuum. In addition, local geometric discontinuities at nodes, surface micro-cracks, and microscopic pores were neglected. The material was assumed to exhibit linear elastic behaviour within the loading range considered in this study, without accounting for plasticity, damage evolution, or complex failure mechanisms.

It should be noted that the present finite element model was developed to characterize the elastic deformation behaviour of sugarcane stalks before structural failure. Plastic deformation, damage accumulation, and fracture processes were not included in this model. Therefore, the model is not intended to predict failure initiation or post-failure behaviour. Furthermore, due to the lack of independently measured anisotropic tensile, compressive, and shear strength data, no anisotropic failure criterion was incorporated into the model. Consequently, the current simulation framework cannot predict damage initiation, crack propagation, or final failure under any loading conditions. Accordingly, the evaluation of the present model is limited to the macroscopic pre-failure response under the quasi-static three-point bending conditions examined in this study, and no predictive capability is claimed for other loading configurations, dynamic processes, or damage and failure behaviour.

#### 2.3.2 Three-dimensional model development and material property assignment

Based on the experimentally measured geometric dimensions of the sugarcane stalks, a three-dimensional solid model was developed using SolidWorks, as shown in **Figure 8**, and subsequently imported into ANSYS Workbench for finite element analysis. To ensure consistency between the numerical model and the experimental conditions, the geometric dimensions, support span, and loading position of the model were defined in strict accordance with the three-point bending test configuration.

**Figure 8:**
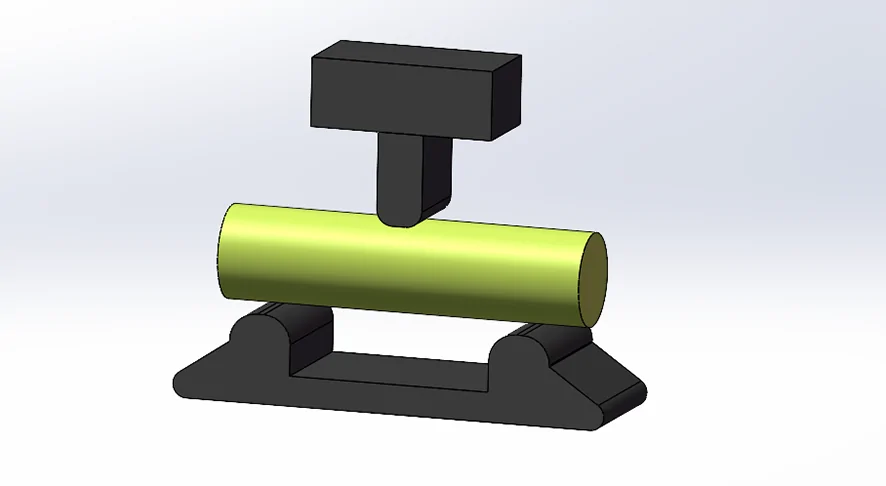
Three-dimensional solid modelling of the sugarcane stalk.

The transversely isotropic material model was primarily defined using the experimentally measured axial elastic modulus (E_z_) and the equivalent radial deformation parameter (E_r_). In the homogenised macroscale model, Er was used as an effective transverse stiffness parameter rather than interpreted as an intrinsic transverse Young’s modulus. The equivalent bending response parameter (E_b_) was not assigned as an independent constitutive input and was used only to characterise the experimental bending response. The material parameters used in the finite element model were obtained from different sources. The axial elastic modulus (E_z_) and equivalent radial deformation parameter (E_r_) were obtained from the present mechanical tests. The Poisson’s ratios and density were adopted from published values because they were not directly measured in the present experiments, while the longitudinal–transverse shear modulus (G_rz_) was calculated using Equation 8. The equivalent bending response parameter (E_b_) was not assigned as an independent constitutive input and was used only to characterise the experimental bending response. Therefore, the finite element model should be interpreted as a macroscale equivalent model for predicting the overall deformation behaviour of sugarcane stalks, rather than a microscale representation of the actual anatomical structure. The simplification of internal tissues allows the model to maintain computational efficiency while preserving the dominant directional mechanical characteristics of sugarcane stalks.

The longitudinal direction of sugarcane internodes is defined as the principal material z-axis, and planes perpendicular to the z-axis are transversely isotropic x-y planes. A cylindrical local coordinate system is adopted in ANSYS for all material parameter assignments. Parameter self-consistency verification is implemented following the Poisson reciprocal rule of transversely isotropic elasticity. All elastic constants can guarantee a positive definite compliance matrix and meet the physical admissibility of materials. The complete symmetric positive definite compliance matrix is presented in Equation 7.

(7) $$\begin{array}{c} \left[\begin{array}{c} \epsilon_{r}\\ \epsilon_{t}\\ \epsilon_{z}\\ \gamma_{rz}\\ \gamma_{rt} \end{array}]=[\begin{array}{ccccc} \frac{1}{E_{r}} & -\frac{\mu_{r}}{E_{r}} & -\frac{\mu_{zr}}{E_{z}} & 0 & 0\\ -\frac{\mu_{r}}{E_{r}} & \frac{1}{E_{r}} & -\frac{\mu_{zr}}{E_{z}} & 0 & 0\\ -\frac{\mu_{zr}}{E_{z}} & -\frac{\mu_{zr}}{E_{z}} & \frac{1}{E_{z}} & 0 & 0\\ 0 & 0 & 0 & \frac{1}{G_{rz}} & 0\\ 0 & 0 & 0 & 0 & \frac{2(1+\mu_{r})}{E_{r}} \end{array}][\begin{array}{c} \sigma_{r}\\ \sigma_{t}\\ \sigma_{z}\\ \tau_{rz}\\ \tau_{rt} \end{array}\right] \end{array}$$

Compared with the conventional approach that directly adopts isotropic material properties, the transversely isotropic parameter assignment method based on experimentally measured data used in this study can more realistically represent the axially reinforced structural characteristics of fibrous tissues within sugarcane stalks, thereby improving the capability of the model to capture actual mechanical behaviour. The material parameters used in the finite element model of the sugarcane stalk are listed in **Table 1**.

**Table 1:** Experimentally measured mechanical parameters and finite element input parameters of sugarcane stalks.

Parameter	Value	Unit	Source
Basal equivalent bending response parameter (E_b_1__)	174.10	MPa	From present three-point bending tests
Middle equivalent bending response parameter (E_b_2__)	140.39	MPa	From present three-point bending tests
Basal equivalent radial deformation parameter (E_r_1__)	17.66	MPa	From present radial compression tests
Basal axial elastic modulus (E_z_1__)	96.95	MPa	From present axial compression tests
Middle equivalent radial deformation parameter (E_r_2__)	14.47	MPa	From present radial compression tests
Middle axial elastic modulus (E_z_2__)	65.90	MPa	From present axial compression tests
Transverse surface Poisson’s ratio (μ_r_)	0.25	/	Literature (Luo et al., 2017)
Basal transverse Poisson’s ratio(μ_zr1_)	0.20	/	Literature (Luo et al., 2017)
Middle transverse Poisson’s ratio(μ_zr2_)	0.20	/	Literature (Luo et al., 2017)
Basal shear modulus(G_rz1_)	40.40	MPa	Calculated via Equation 8
Middle shear modulus(G_rz2_)	27.46	MPa	Calculated via Equation 8
Density (ρ)	1190	(kg·m^−3^)	Literature (Ren et al., 2022)
Length	80	(mm)	Mean geometric dimensions of experimental specimens

The three-point bending specimens used for comparison with the finite element simulations were not part of a separately collected independent validation dataset. They were the same bending specimens included in the experimental programme described above. These specimens were physically separate from those used for the axial and radial compression tests, from which the experimentally informed axial and transverse stiffness parameters were obtained, but all specimens originated from the same overall collection of sugarcane stalks. In addition, the equivalent bending response parameter (E_b_) derived from the three-point bending tests was not assigned as an independent constitutive input to the finite element model. Accordingly, the comparison with the three-point bending experiments was used to assess whether the macroscale model could reproduce the global pre-failure bending response under the present quasi-static loading configuration, rather than as an independent validation of general predictive capability.

The longitudinal-transverse shear modulus G_rz_ is calculated by the standard linear elastic formula, as shown in Equation (8):

(8) $$G_{rz}=\frac{E_{z}}{2(1+\mu_{zr})}$$

#### 2.3.3 Mesh generation and mesh independence analysis

To ensure numerical accuracy and computational efficiency, the three-dimensional sugarcane stalk model was discretised using ANSYS Workbench. Considering the stress concentration characteristics at loading and supporting positions under three-point bending, a hybrid meshing strategy was adopted in this study. The global model was discretised with hexahedron-dominant meshes, while local mesh refinement was specifically implemented at the indenter–stalk contact region and the two support–stalk contact regions to accurately capture local stress gradients and contact deformation. Hexahedron-dominant meshes provide better numerical stability and convergence behaviour for bending problems compared with tetrahedral meshes, which is more suitable for the mechanical simulation of slender plant stalk structures.

A systematic mesh independence analysis was performed to eliminate the influence of mesh density on numerical results. Four global mesh sizes (3.0 mm, 2.0 mm, 1.0 mm, and 0.5 mm) were tested under identical boundary conditions, contact settings, and loading parameters. Notably, the locally refined mesh size at contact interfaces remained consistent in all groups to guarantee a fair comparison. The total element number, node number, mechanical responses, relative errors, and computational time for each mesh scheme are summarised in **Table 2**.

**Table 2:** Mesh convergence results of typical mesh schemes.

Global mesh size (mm)	Total elements	Total nodes	Displacement relative error (%)	Stress relative error (%)	Reaction relative error (%)	Calculation time (min)
3.0	5386	21462	+6.72	+8.35	+5.76	1.1
2.0	9741	39105	+2.01	+3.92	+2.48	2.6
1.0	18467	75892	−2.07	−0.51	+0.62	6.2
0.5	31852	126937	0.00	0.00	0.00	14.5

In this study, the numerical results obtained from the finest mesh (0.5 mm) were regarded as the converged reference solution for relative error calculation. Positive relative error values indicate that the predicted result of a coarse mesh is higher than the converged value, whereas negative values indicate that the predicted result is slightly lower than the converged solution.

As the global mesh size gradually reduced from 3.0 mm to 0.5 mm, the calculated maximum mid-span displacement, maximum equivalent stress and support reaction force exhibited a gradual convergence trend. For the 3.0 mm coarse mesh, the relative errors of displacement, maximum equivalent stress and support reaction force reached 6.72%, 8.35% and 5.76%, respectively. When the global mesh was refined to 2.0 mm, all relative errors decreased obviously, with values of 2.01%, 3.92% and 2.48%. Further reducing the global mesh size to 1.0 mm, the relative errors of the three indicators dropped to −2.07%, −0.51% and 0.62%. After further mesh refinement from 1.0 mm to 0.5 mm, the change range of all key indicators was controlled within 2.1%, satisfying the commonly adopted convergence criterion that the relative error should be less than 3% for finite element simulation of biomass materials. Although the 0.5 mm mesh achieves the highest calculation accuracy, the total number of elements increases significantly, and the computational time rises sharply to 14.5 min, which greatly reduces computational efficiency.

Considering both computational accuracy and solution efficiency, a global mesh size of 1.0 mm was ultimately selected for the finite element model of the sugarcane stalk, as shown in **Figure 9**. This mesh configuration can adequately capture the stress distribution and deformation characteristics of the sugarcane stalk under bending loads, while also meeting the requirements for subsequent model evaluation and parameter analysis.

**Figure 9:**
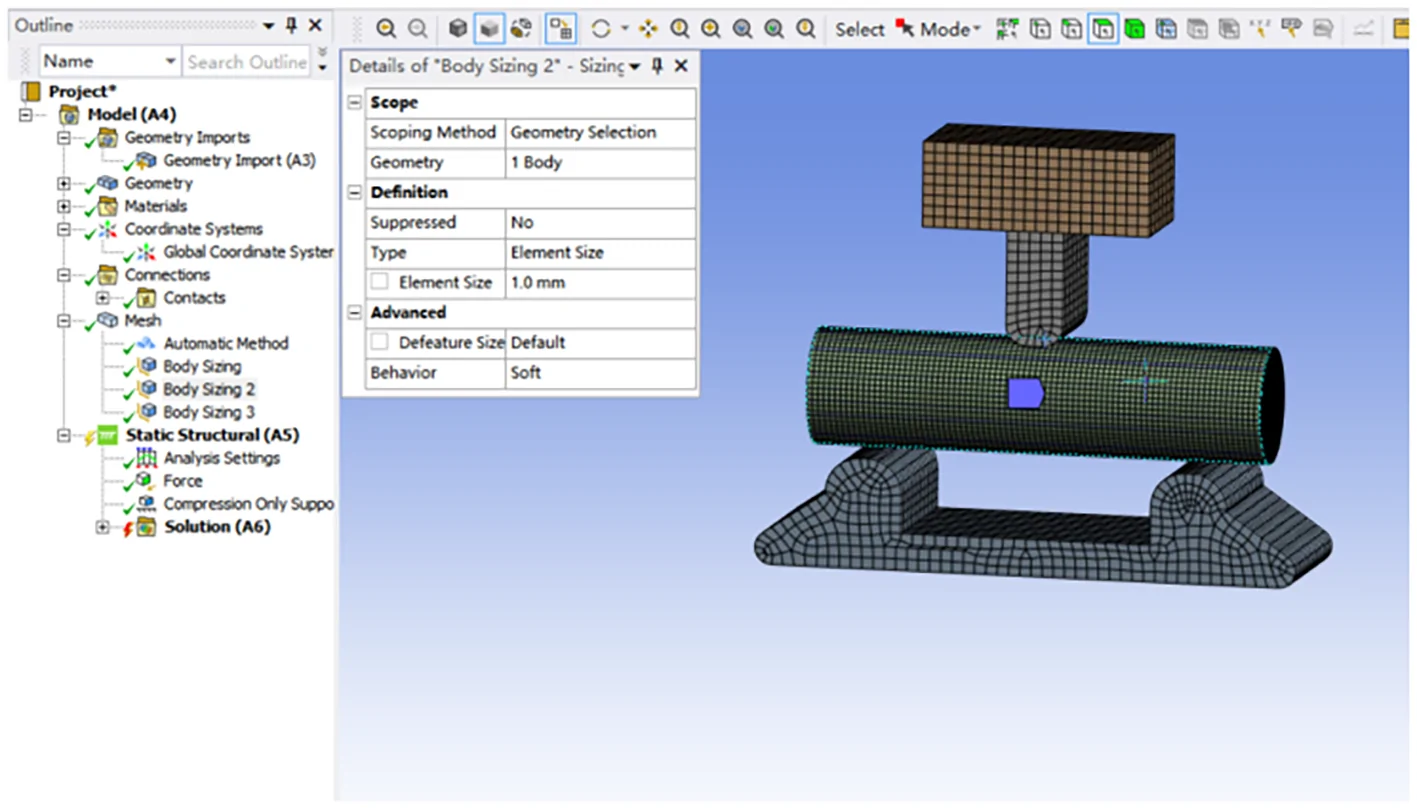
Minimum element size of the finite element mesh for sugarcane stalks.

#### 2.3.4 Boundary conditions and loading conditions

To assess the ability of the transversely isotropic finite element model to reproduce the experimental response under three-point bending, the boundary conditions were defined to correspond to the experimental configuration. During the experiment, the sugarcane specimen was fully restrained by supports without horizontal sliding or rolling throughout loading, and only vertical axial load existed with negligible tangential relative motion. Therefore, all translational and rotational degrees of freedom of the two support rigid reference points were fully fixed. All of the loading indenter were locked except the vertical displacement direction for displacement-controlled loading. The total vertical displacement was limited to 10 mm with a uniform loading speed of 20 mm/min to reproduce quasi-static bending deformation and the loading devices during the three-point bending process, as shown in **Figure 10**.

**Figure 10:**
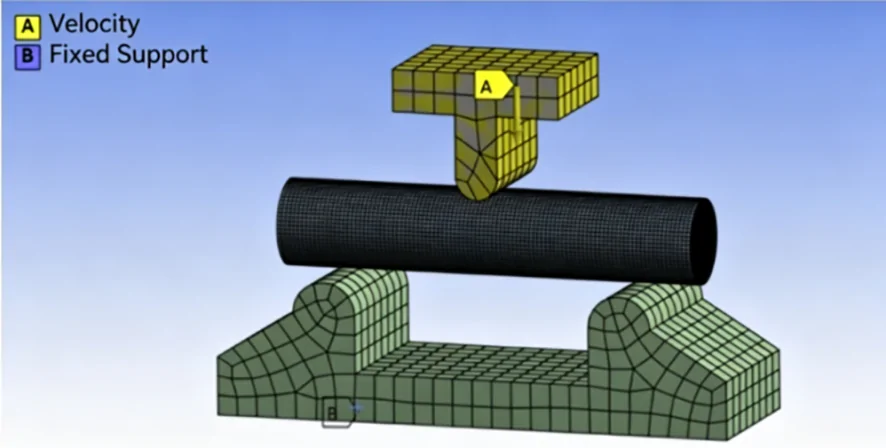
Boundary condition setup.

The simulation outputs included load–displacement curves, maximum mid-span displacement, support reaction forces, and von Mises stress contour distributions. The macroscopic load and displacement responses were compared with the experimental results, whereas the internal stress and strain fields were treated as numerical predictions of the finite element model. The von Mises stress distribution was used only to visualise the model-predicted stress concentration during bending deformation and was not experimentally validated. Since the current model assumes an equivalent transversely isotropic elastic material and does not incorporate anisotropic failure criteria, the stress contours should not be interpreted as direct evidence of tissue damage or fracture initiation.

### 2.4 Data processing and statistical analysis

To ensure the reliability and comparability of the experimental results, statistical analysis and significance testing were conducted on the mechanical parameters of sugarcane stalks. The mean value, standard deviation, standard error, and coefficient of variation were used to describe the central tendency and dispersion of each parameter. For a given mechanical parameter with n replicate specimens, the mean value (*x̄*), standard deviation (*s*), standard error (*SE*), and coefficient of variation (*CV*) are defined as follows, using Equations (9–12):

(9) $$\bar{x}=\frac{1}{n}\overset{n}{\underset{i=1}{\sum }}x_{i}$$

(10) $$s=\sqrt{\frac{1}{n-1}\sum_{i=1}^{n}{\left(x_{i}-\bar{x}\right)}^{2}}$$

(11) $$SE=\frac{s}{\sqrt{n}}$$

(12) $$CV=\frac{s}{\bar{x}}\times 100\%$$

where *x_i_* is the mechanical parameter value of the i-th specimen, and *n* is the number of replicate samples. The mean value is used to characterise the overall level of the parameter, while the standard deviation and standard error describe the variability of the samples and the stability of the mean estimation, respectively. The coefficient of variation is used to compare the relative dispersion among different parameters. To compare the mechanical properties between the basal and middle sections, measurements obtained from the same sugarcane stalk were treated as paired observations. Two-tailed paired-samples t-tests were therefore used for the axial elastic modulus (E_z_), equivalent radial deformation parameter (E_r_), and equivalent bending response parameter (E_b_). The normality of the within-stalk paired differences was assessed using the Shapiro–Wilk test before the paired-samples t-test. Statistical significance was set at p < 0.05. Statistical analyses, except for the *post-hoc* power analysis, were performed using Origin software, and the level of significance was set at *p* < 0.05. The results are presented as mean ± standard deviation (Mean ± *SD*).

Cohen’s d_z_ was calculated to quantify the effect size of the paired basal–middle difference. Cohen’s d_z_ was defined as the mean of the within-stalk paired differences divided by the standard deviation of these paired differences. According to conventional criteria, values of 0.2, 0.5, and 0.8 were considered small, medium, and large effects, respectively using Equation (13):

(13) $$dz=\frac{D}{\text{S}D}$$

where is Cohen’s effect size for paired data, D is the mean of the within-stalk paired differences between the basal and middle sections, and is the standard deviation of these paired differences.

To analyse the influence of geometric dimensions on the mechanical properties of sugarcane stalks, linear regression analysis was further performed between the length and diameter of the stalks and each mechanical parameter. The coefficient of determination (*R²*) was used to evaluate the extent to which geometric dimensions explain the variation in these parameters. The regression model is expressed as Equation (14):

(14) y=∂x+β

where *y_i_* is the measured value, *ŷ_i_* is the corresponding fitted (predicted) value, and *ȳ* is the sample mean. A higher *R²* value indicates a stronger explanatory ability of the corresponding geometric dimension for the variation in the mechanical parameter; conversely, a lower *R²* value suggests a weaker correlation between them.

In the experimental comparison of the finite element model, peak load, peak displacement, and their relative errors were used to assess the agreement between the simulated and experimental bending responses. The relative error is defined as Equation (15):

(15) $$\delta =\frac{\left|X_{\text{sim}}-X_{\exp}\right|}{X_{\exp}}\times 100\%$$

where *X_sim_* denotes the simulation result and *X_exp_* denotes the experimental result. A smaller value of *δ* indicates that the simulated results are in good agreement with the experimental results for the corresponding indicator. In addition to the numerical error metrics, agreement in the load–displacement response was also considered in evaluating the finite element model. Agreement between the simulated and experimental curves was used to assess whether the model could reasonably reproduce the overall pre-failure bending response of sugarcane stalks under the present three-point bending conditions. In the statistical analysis, the individual sugarcane stalk was defined as the biological replicate and primary statistical unit. Basal and middle measurements obtained from the same stalk were therefore treated as paired observations. Repeated specimens obtained from the same stalk were not considered additional independent biological replicates. The effective number of independent stalk-level pairs was reported separately for each mechanical test: n = 20 pairs for the axial compression analysis, n = 20 pairs for the radial compression analysis, and n = 10 pairs for the three-point bending analysis.

For the regression analysis, the 95% confidence intervals for the prediction values were calculated based on the standard error of the estimate, using Equations (16, 17):

(16) $${\hat{y}}_{0}\pm t_{\alpha /2,n-2}\cdot s_{e}\cdot \sqrt{\frac{1}{n}+\frac{{\left(x_{0}-\bar{x}\right)}^{2}}{S_{xx}}}$$

(17) $$S_{xx}=\sum {\left(x_{i}-\bar{x}\right)}^{2}$$

The mechanical anisotropy of sugarcane stalks was quantified by calculating the anisotropy ratio, defined as the ratio of axial elastic modulus to equivalent radial deformation parameter $\left(E_{z}/E_{r}\right)$.

The 95% confidence intervals (CIs) for the mean values were calculated using the t-distribution to account for the relatively small sample sizes, using Equation (18):

(18) $$CI_{95\%}=\bar{x}\pm t_{0.975,n-1}\times \frac{SD}{\sqrt{n}}$$

where $\bar{x}$ is the sample mean, $t_{0.975,n-1}$ is the critical t-value with n−1degrees of freedom, SD is the standard deviation, and n is the sample size.

For the regression analysis, the 95% CIs for the prediction values were calculated based on the standard error of the estimate, following Equation (19):

(19) $${\hat{y}}_{0}\pm t_{\alpha /2,n-2}\cdot s_{e}\cdot \sqrt{\frac{1}{n}+\frac{{\left(x_{0}-\bar{x}\right)}^{2}}{S_{xx}}}$$

Where ${\hat{y}}_{0}$ is the predicted value, $t_{\alpha /2,n-2}$is the critical t-value with n−2 degrees of freedom at α =0.05 α=0.05, $S_{e}$ is the standard error of the estimate, and $S_{xx}=\sum {\left(x_{i}-\bar{x}\right)}^{2}$.

## 3 Results and analysis

### 3.1 Experimental design and evaluation indicators

To systematically investigate the mechanical properties of sugarcane stalks and the applicability of the developed finite element model, this chapter presents analyses from three aspects: mechanical parameter characteristics, size effects, and experimental comparison. Firstly, based on the results of axial compression, radial compression, and three-point bending tests, the variations in axial elastic modulus (E_z_), equivalent radial deformation parameter (E_r_), and equivalent bending response parameter (E_b_) are analysed. Differences between specimens from different internodal positions are compared, and the anisotropic mechanical behaviour of sugarcane stalks is evaluated. Secondly, correlation analysis is conducted to examine the relationship between specimen geometric dimensions and mechanical parameters, in order to reveal the influence of size-related factors on mechanical performance. Finally, a finite element model is established based on the experimentally obtained mechanical parameters, and its ability to reproduce the measured response was assessed by comparing numerical simulation results with experimental data.

In the analysis of results, the mean (Mean) and standard deviation (*SD*) were used to describe the statistical characteristics of the mechanical parameters, while the coefficient of variation (*CV*) was employed to evaluate the degree of data dispersion. Linear regression analysis was conducted to investigate the relationships between geometric dimensions and mechanical parameters, and the coefficient of determination (*R²*) was used to assess the goodness of fit of the regression models. Paired-samples t-tests were applied to examine differences in mechanical parameters between the basal and middle sections, with measurements from the same stalk treated as paired observations. In addition, the agreement between the simulated and experimental load–displacement curves was considered to comprehensively assess the agreement with experimental measurements of the model.

Based on the above analytical framework, the subsequent sections will sequentially present and discuss the results of axial compression behaviour, radial compression behaviour, three-point bending behaviour, and finite element experimental comparison.

### 3.2 Axial compression results and analysis

#### 3.2.1 Comparison of axial elastic modulus between basal and middle regions

The statistical results of the axial elastic modulus (E_z_) for the basal and middle sugarcane stalk specimens are presented in **Figure 11**, **Tables 3**, **4**. The descriptive statistics, 95% confidence intervals (CIs), and significance test results for the axial elastic modulus are presented in **Table 5**. The results show that the mean axial elastic modulus of the basal specimens is 96.95 MPa (95% CI: 85.07–108.83 MPa), with a standard deviation of 25.39 MPa, a standard error of 5.68 MPa, and a coefficient of variation of 26.2%. For the middle specimens, the mean axial elastic modulus is 65.90 MPa (95% CI: 58.61–73.19 MPa), with a standard deviation of 15.58 MPa, a standard error of 3.48 MPa, and a coefficient of variation of 23.6%.

**Figure 11:**
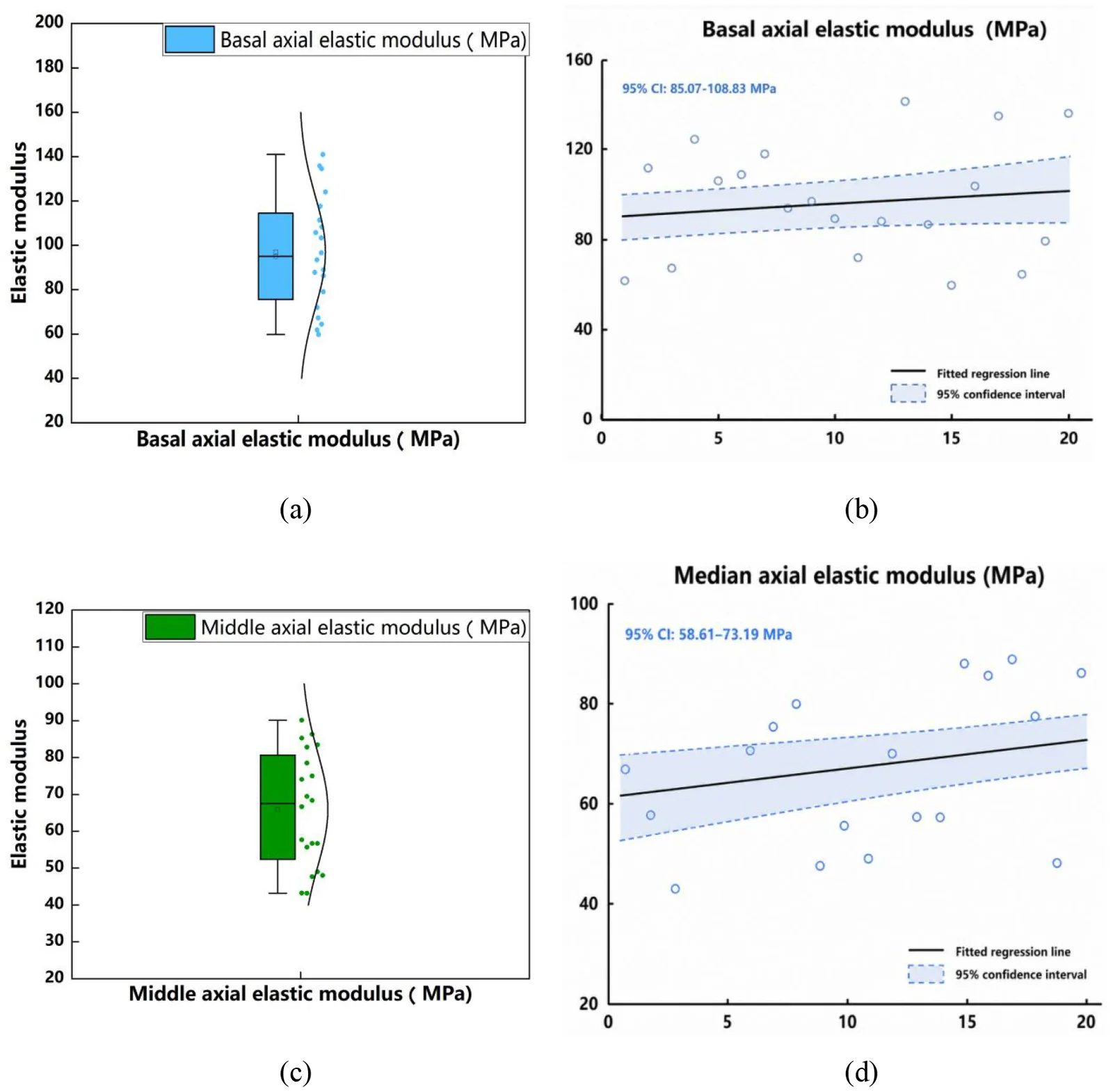
Statistical distribution of axial elastic modulus (E_z_) of basal and middle sugarcane stalks (n = 20).

**Table 3:** Statistical results of axial elastic modulus for basal specimens.

No.	Stalk length (mm)	Stalk diameter (mm)	Axial elastic modulus at base (MPa)
1	60.2	19.7	61.82
2	63.6	20.1	111.36
3	63.27	22.1	67.26
4	63.43	24.6	124.07
5	64.8	21.4	105.72
6	68.2	20.1	108.29
7	70.88	21.3	117.59
8	71.3	23	93.47
9	71.77	24.5	96.59
10	72.6	26.2	88.95
11	72.7	24.8	71.93
12	73.23	24	87.78
13	74.67	23.7	140.97
14	77	21.3	86.24
15	77.77	28.9	59.79
16	78.8	25.1	103.34
17	83.9	23.7	134.55
18	75.8	23.7	64.40
19	85.96	27.8	79.10
20	88	24.6	135.75

**Table 4:** Statistical results of axial elastic modulus for middle specimens.

No.	Stalk length (mm)	Stalk diameter (mm)	Middle-section axial elastic modulus (MPa)
1	46.6	24.6	66.65
2	47.1	24.8	57.68
3	49.63	23.9	43.245
4	52.7	23.6	43.17
5	57.47	22.3	90.16
6	58.3	23.6	69.47
7	58.3	23.9	74.07
8	59	26.1	78.51
9	60.13	27.8	47.67
10	62.77	24.2	55.67
11	63.67	23.4	49.01
12	64.37	25.7	68.36
13	65.6	25.9	56.69
14	65.23	29.7	56.7
15	66.83	26.3	85.28
16	67.23	24.1	82.82
17	68	25.5	86.34
18	69.5	25.7	75.02
19	78.93	28.1	48.02
20	87.73	22.1	83.44

**Table 5:** Statistical comparison of axial elastic modulus between basal and middle sections.

Parameter	Section	Mean ± SD (MPa)	95%CI (MPa)	t	d_f_	p	Cohen’s dz
Axial elastic modulus (E_z_)	Basal	96.95 ± 25.39	85.07-108.83	5.08	19	<0.001	1.14
	Middle	65.90 ± 15.58	58.61-73.19	**-**	**-**	**-**	**-**

A two-tailed paired-samples t-test was further conducted to compare the axial elastic modulus between the basal and middle regions. Cohen’s dz was calculated to quantify the effect size of the paired difference. The results showed a statistically significant difference between the two regions. The axial elastic modulus of the basal region was approximately 47.12% higher than that of the middle region, indicating greater resistance to axial compressive deformation in the basal portion.

The basal specimens exhibited a significantly higher axial elastic modulus than the middle specimens, indicating a clear longitudinal difference in axial mechanical response under the present experimental conditions. This difference may be associated with longitudinal variations in internal tissue organisation. However, vascular bundle distribution, fibre content, lignification, and related anatomical characteristics were not directly quantified in this study. Therefore, these factors should be regarded as possible explanations rather than experimentally demonstrated mechanisms.

Both the basal and middle specimens also showed a certain degree of data dispersion, reflecting specimen-to-specimen variability. Because moisture content and anatomical characteristics were not directly measured, the sources of this variability could not be quantitatively determined. Overall, the axial compression results demonstrate a clear difference in axial stiffness between the tested basal and middle specimens and provide the experimental axial stiffness parameters used in the subsequent transversely isotropic finite element modelling.

#### 3.2.2 Correlation analysis between geometric dimensions and axial elastic modulus

To further analyse the influence of specimen geometry on the axial elastic modulus, linear regression relationships between specimen length and diameter and the axial elastic modulus were established for the basal and middle sections, respectively. The results are shown in **Figure 12**.

**Figure 12:**
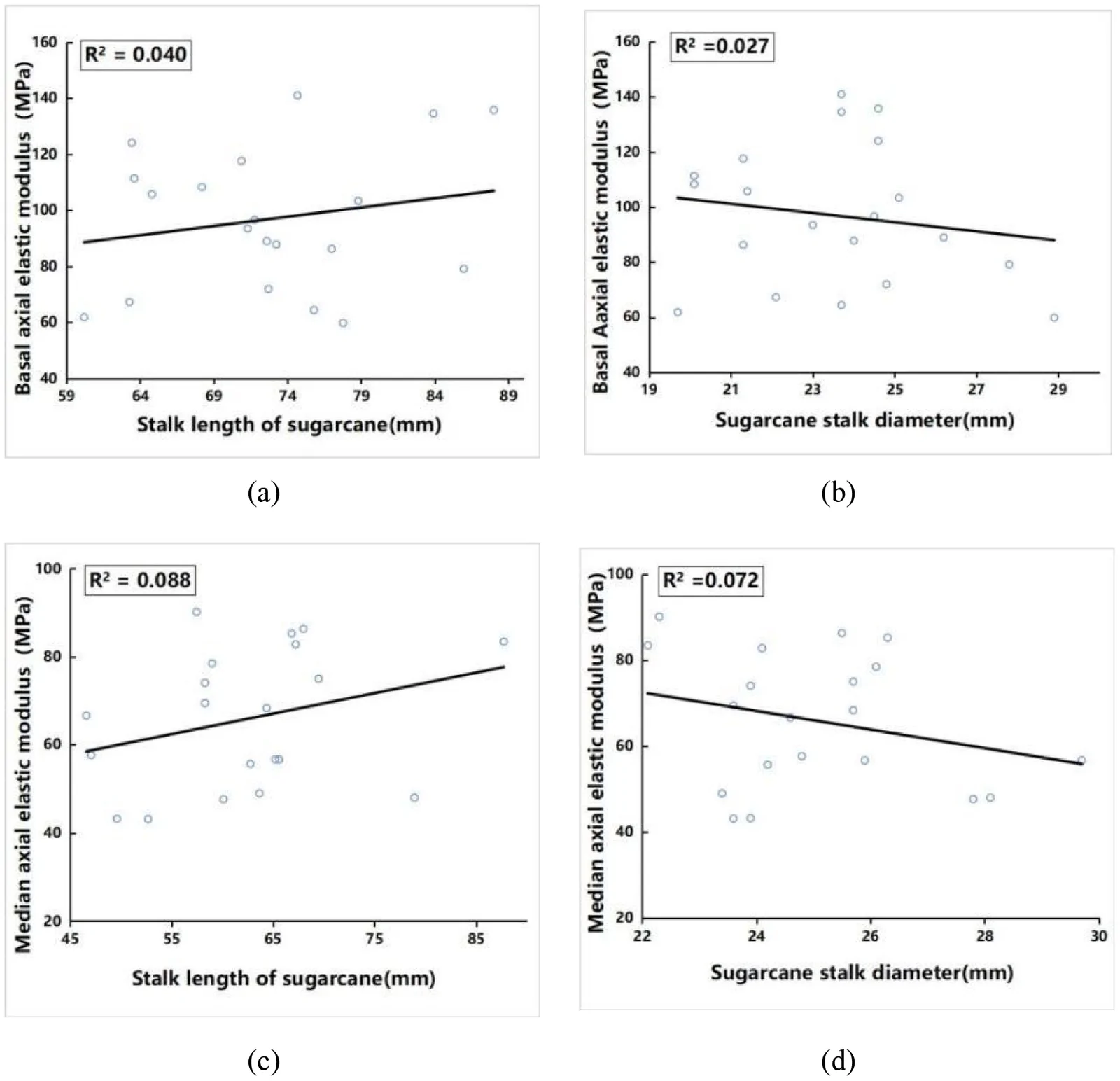
Coefficient of determination (*R²*) for the relationships between sugarcane stalk length and diameter and axial elastic modulus (E_z_).

The results indicate that, for basal specimens, the coefficient of determination *R^2^* between length and axial elastic modulus is relatively low, and only a weak linear correlation was observed. Similarly, the correlation between diameter and axial elastic modulus in the basal specimens is weak. For the middle-section specimens, the *R^2^* value between length and axial elastic modulus is also at a low level, and only a weak linear correlation was observed between diameter and axial elastic modulus. Overall, the regression results exhibit considerable scatter across all groups, suggesting that specimen geometry has limited explanatory power and is not the dominant factor governing variations in axial elastic modulus.

The weak correlations indicate that the measured specimen length and diameter had limited explanatory power for the variation in axial elastic modulus. However, these results alone do not identify the internal anatomical factors responsible for this variation. Therefore, anatomical characteristics such as vascular bundle distribution and fibre content should not be regarded as experimentally demonstrated mechanisms in the present study.

In addition, natural plant materials are influenced during growth by multiple factors, including cultivar, maturity, moisture condition, and environmental conditions, resulting in a highly stochastic internal tissue structure. This structural heterogeneity further weakens the linear relationship between geometric dimensions and mechanical parameters. From the perspective of finite element modelling, these findings suggest that, when constructing mechanical models of sugarcane stalks, relying solely on geometric dimensions for parameter homogenisation has inherent limitations, whereas the introduction of direction-dependent elastic parameters can more realistically represent the characteristics of the internal fibre-reinforced structure. Therefore, in subsequent development of transversely isotropic finite element models, it is necessary to place emphasis on the differences in mechanical properties along different directions, rather than simplifying the representation based only on external geometric parameters.

### 3.3 Radial compression test results and analysis

#### 3.3.1 Comparison of statistical results of equivalent radial deformation parameter between basal and middle sections

The statistical results of the equivalent radial deformation parameter (E_r_) for basal and middle sugarcane stalk specimens are shown in **Figure 13**, **Tables 6**, **7**. The descriptive statistics, 95% confidence intervals (CIs), and statistical comparison results for the equivalent radial deformation parameter are summarized in **Table 8**. The mean equivalent radial deformation parameter of the basal specimens was 17.66 MPa (95% CI: 15.90–19.42 MPa), with a standard deviation of 3.76 MPa, a standard error of 0.84 MPa, and a coefficient of variation of 21.30%. For the middle-section specimens, the mean value was 14.47 MPa (95% CI: 12.58–16.35 MPa), with a standard deviation of 4.03 MPa, a standard error of 0.90 MPa, and a coefficient of variation of 27.80%. The lower CV of the basal specimens indicates lower relative variability than that observed in the middle section.

**Figure 13:**
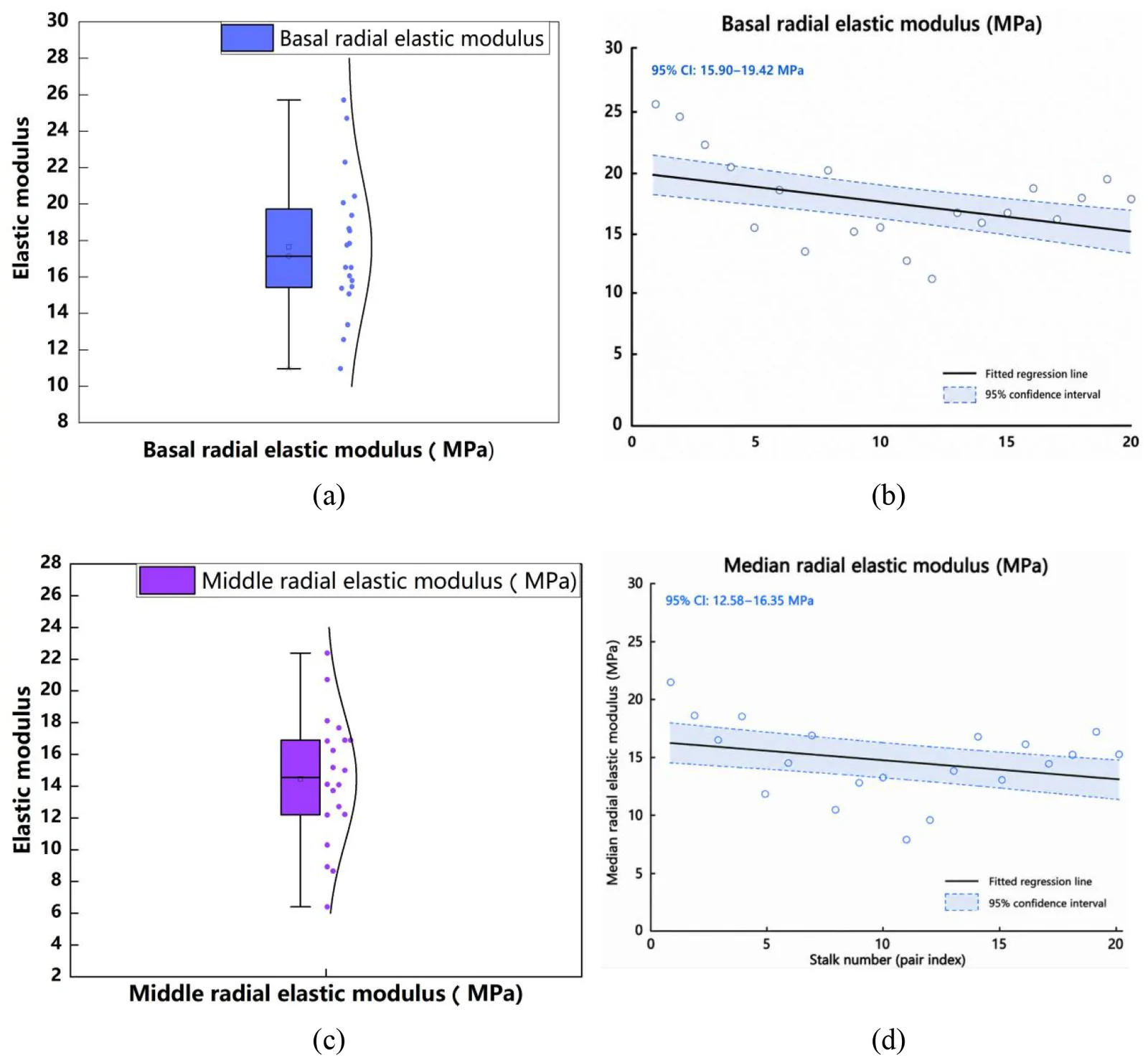
Statistical distribution of equivalent radial deformation parameter (E_r_) of basal and middle sugarcane stalks (n = 20).

**Table 6:** Statistical results of equivalent radial deformation parameter for basal specimens.

No.	Stalk length (mm)	Stalk diameter (mm)	Equivalent radial deformation parameter at base (MPa)
1	58.9	20.6	25.71
2	70.33	24.5	24.71
3	72.2	24.8	22.30
4	74.73	23.3	20.43
5	75.5	22.9	15.38
6	77	22.8	18.53
7	77.23	25.4	13.38
8	78.57	27.6	20.07
9	78.83	27.6	15.07
10	85.47	23.4	15.47
11	85.53	22.5	12.56
12	85.67	27.5	10.97
13	86.37	23.8	16.52
14	88.77	21.6	15.8
15	89.37	26.6	16.53
16	91.97	23.6	18.66
17	95.77	23.3	16.06
18	98.17	26.8	17.84
19	101.03	23.9	19.38
20	103.02	26.4	17.75

**Table 7:** Statistical results of equivalent radial deformation parameter for middle-section specimens.

No.	Stalk length (mm)	Stalk diameter (mm)	Equivalent radial deformation parameter at middle (MPa)
1	51.83	23.8	12.19
2	52.53	23.5	10.30
3	55.67	27.1	16.85
4	55.3	26.5	20.72
5	56	23.7	16.25
6	58.8	25	6.40
7	58.13	27.1	22.39
8	64.53	27.7	18.12
9	65.07	27.6	14.12
10	67.63	27.08	17.68
11	67.93	24.4	8.92
12	68.7	24	16.90
13	69	24.1	15.18
14	69.7	24.9	13.73
15	72.51	24.1	12.71
16	73.03	24.4	14.09
17	77.3	27.6	15.00
18	79.1	25.1	12.22
19	80.5	23.6	8.66
20	58.8	27.3	16.9

**Table 8:** Statistical comparison of equivalent radial deformation parameter between basal and middle sections.

Parameter	Section	Mean ± SD (MPa)	95%CI (MPa)	t	d_f_	p	Cohen’s dz
equivalent radial deformation parameter (Er)	Basal	17.66 ± 3.76	15.90-19.42	2.36	19	0.029	0.53
	Middle	14.47 ± 4.03	12.58-16.35	**-**	**-**	**-**	**-**

A two-tailed paired-samples t-test was conducted to compare the equivalent radial deformation parameter between the basal and middle sections. Cohen’s was calculated to quantify the effect size of the paired difference. The results showed a statistically significant difference between the two regions. The mean equivalent radial deformation parameter of the basal section was approximately 22.05% higher than that of the middle section, indicating a significant longitudinal difference in radial deformation response along the sugarcane stalk.

Unlike axial loading, radial compression involves the application of load perpendicular to the alignment of vascular bundles, under which the reinforcing effect of fibrous vascular bundles is significantly reduced. In this case, parenchyma cells and pith tissues may become the primary load-bearing components. Under radial compression, the internal parenchyma tissues may undergo progressive deformation, potentially involving cell wall buckling and collapse (Speck and Burgert, 2011; Niklas, 2013), which could contribute to the lower apparent stiffness observed in the radial direction. Therefore, radial mechanical properties may be influenced not only by fibrous tissues but also by the proportion of pith tissue and the structural stability of cellular components (Speck and Burgert, 2011), although these anatomical factors were not directly quantified in the present study.

The results of this study indicate that the equivalent radial deformation parameter of basal specimens is significantly higher than that of middle-section specimens (p < 0.05). This difference may be associated with longitudinal variation in internal tissue organisation. However, vascular bundle distribution, fibre content, and related anatomical characteristics were not directly quantified in the present study. Therefore, these structural factors should be regarded as possible explanations rather than experimentally demonstrated mechanisms.

In addition, due to the presence of pore structures and tissue heterogeneity within natural biological materials, the stress distribution during radial compression is often non-uniform, and localised stress concentrations are likely to occur. As compressive deformation increases, irreversible damage to cellular structures develops, leading to a further reduction in overall stiffness. The equivalent radial deformation parameter being significantly lower than the axial elastic modulus indicates that sugarcane stalks exhibit markedly different mechanical responses under different loading directions, further confirming their typical transversely isotropic behaviour.

#### 3.3.2 Correlation analysis between geometric dimensions and equivalent radial deformation parameter

To further analyse the influence of geometric dimensions on radial mechanical properties, linear regression relationships between specimen length and diameter and equivalent radial deformation parameter were established for both basal and middle-section specimens, respectively. The results are shown in **Figure 14**.

**Figure 14:**
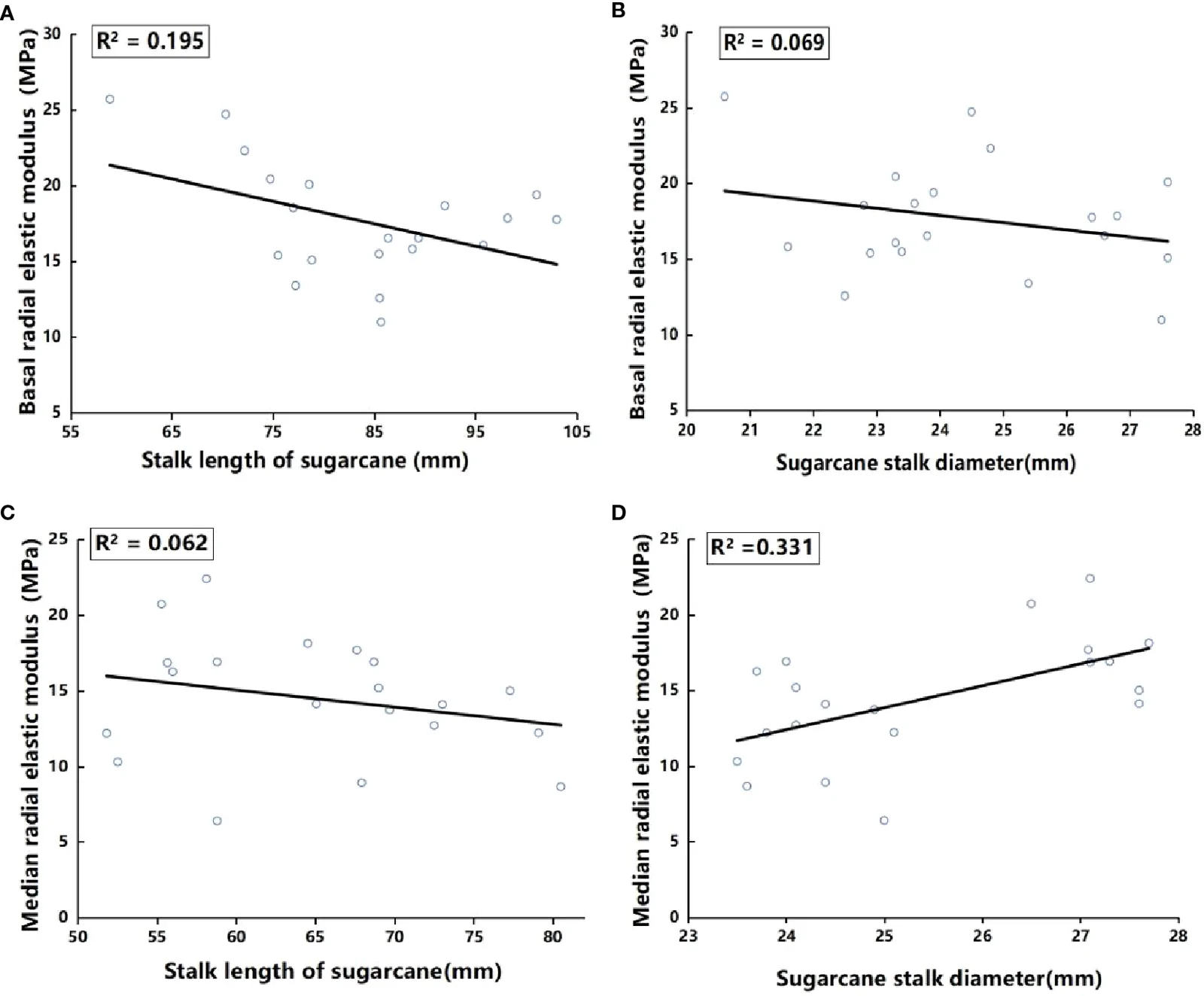
Coefficient of determination (*R²*) for the relationships between sugarcane stalk length and diameter and equivalent radial deformation parameter(Er)(n=20).

For basal specimens, the coefficient of determination (*R²*) between length and equivalent radial deformation parameter is 0.195, while that between diameter and equivalent radial deformation parameter is 0.069. For middle-section specimens, the *R²* values are 0.062 for length and 0.331 for diameter, respectively. Overall, the coefficients of determination across all regression models remain relatively low, indicating that there is no significant linear correlation between macroscopic geometric dimensions and equivalent radial deformation parameter, and that geometric parameters have limited explanatory power for radial mechanical responses. From the distribution characteristics of the results, the correlation between diameter and equivalent radial deformation parameter in the middle section is relatively higher; however, it still does not reach a level suitable for reliable engineering prediction. This suggests that cross-sectional dimensions may have a certain influence on stress distribution during local contact compression, but are insufficient to establish a stable scale-dependent relationship. These results indicate that the measured geometric variables had limited explanatory power for the observed variation in Er. However, the present analysis does not identify the internal anatomical factors responsible for the remaining variation.

From a microscopic perspective, radial compression of sugarcane stalks is mainly borne by parenchyma cells, pith tissues, and locally distributed vascular bundles. However, due to the absence of a continuous fibre-reinforced structure in the radial direction, the load transfer pathway is less well-defined than in the axial direction, making it more susceptible to the randomness of tissue distribution. Therefore, the present results, supported by both statistical correlation analysis and significance testing, indicate that the radial mechanical properties of sugarcane stalks should be regarded as a “structure-dominated variable” rather than a “geometry-dominated variable”. The significant difference in equivalent radial deformation parameter (E_r_) between basal and middle sections further demonstrates that, in addition to directional dependence, sugarcane stalks also exhibit longitudinal spatial heterogeneity.

### 3.4 Three-point bending test results and analysis

#### 3.4.1 Statistical characteristics and comparative analysis of equivalent bending response parameter between basal and middle sections

The statistical results of the equivalent bending response parameter (E_b_) for basal and middle sugarcane stalk specimens are presented in **Figure 15**, **Tables 9**–**11** summarizes the descriptive statistics, 95% confidence intervals (CIs), and statistical comparison results for the equivalent bending response parameter. The mean equivalent bending response parameter of the basal specimens was 174.10 MPa (95% CI: 154.40–193.80 MPa), with a standard deviation of 27.54 MPa, a standard error of 8.71 MPa, and a coefficient of variation of 15.82%. For the middle-section specimens, the mean value was 140.39 MPa (95% CI: 123.13–157.64 MPa), with a standard deviation of 24.12 MPa, a standard error of 7.63 MPa, and a coefficient of variation of 17.18%. The relatively low CV values indicate limited relative dispersion in the equivalent bending response parameter within both sections.

**Figure 15:**
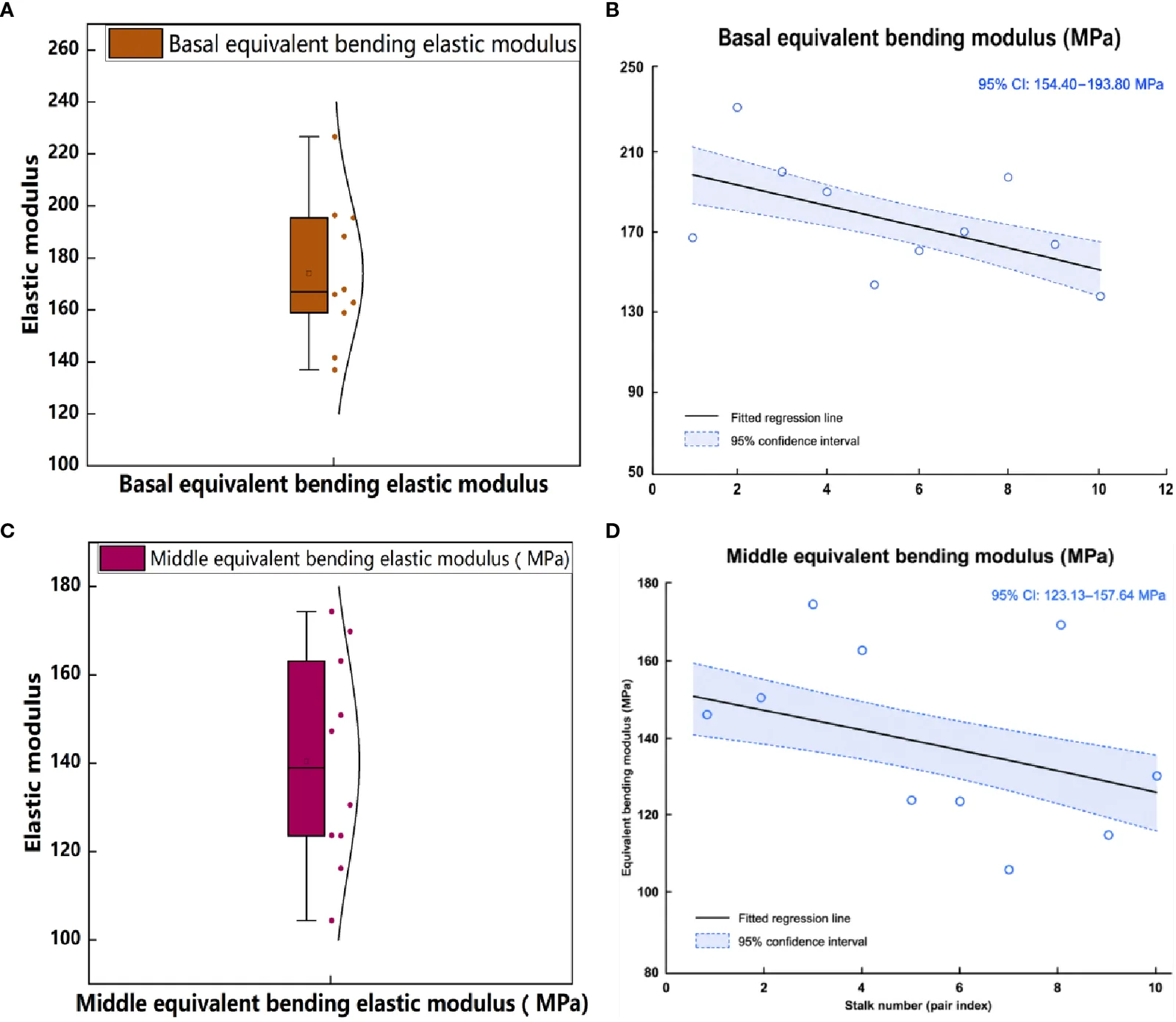
Statistical distribution of equivalent bending response parameter (E_b_) of basal and middle sugarcane stalks (n = 10).

**Table 9:** Statistical results of equivalent bending response parameter for basal specimens.

No.	Stalk diameter (mm)	Load (N)	Equivalent bending response parameter at base(MPa)
1	20.2	847.2	165.96
2	20.3	683.4	226.68
3	21.7	1316	196.47
4	21.9	818	188.27
5	22.1	844.3	141.57
6	22.5	806	158.90
7	22.6	934	167.93
8	23.2	1219	195.46
9	23.8	1106.5	162.82
10	24.8	1654.4	136.91

**Table 10:** Statistical results of equivalent bending response parameter for middle-section specimens.

No.	Stalk diameter (mm)	Load (N)	Equivalent bending response parameter at middle (MPa)
1	21.5	990	147.28
2	21.8	994	150.89
3	23.2	964.6	174.3
4	23.7	1269.2	163.17
5	24.4	974	123.66
6	24.7	1129.2	123.58
7	25.1	1309.1	104.38
8	25.1	1217.2	169.84
9	25.3	1355.7	116.21
10	25.8	1266.4	130.54

**Table 11:** Statistical comparison of equivalent bending response parameter between basal and middle sections.

Parameter	Section	Mean ± SD (MPa)	95%CI (MPa)	t	d_f_	p	Cohen’s dz
equivalent bending response parameter (Eb)	Basal	174.10 ± 27.54	154.40-193.80	4.86	9	<0.001	1.54
	Middle	140.39 ± 24.12	123.13-157.64	**-**	**-**	**-**	**-**

The mean equivalent bending response parameter of the basal section was approximately 24.01% higher than that of the middle section. A two-tailed paired-samples t-test was conducted to compare the basal and middle sections, and Cohen’s was calculated to quantify the effect size of the paired difference. The results showed a statistically significant difference between the two regions, indicating a significant longitudinal difference in bending response along the sugarcane stalk.

The results of this study indicate that the equivalent bending response parameter of basal specimens is significantly higher than that of middle-section specimens (p < 0.05), suggesting a pronounced longitudinal gradient in bending performance along the sugarcane stalk. Compared with the middle section, the basal region exhibits greater overall resistance to bending deformation under the present three-point bending configuration. During three-point bending, the upper surface of the specimen is subjected to compressive stress, while the lower surface is subjected to tensile stress, with a neutral axis forming within the cross-section. Therefore, bending deformation is essentially a combined loading process involving both tension and compression. Unlike radial compression, vascular bundle fibres during bending not only resist compressive loading but also sustain significant tensile loading, allowing their reinforcing effect to be more fully mobilised. The outer region of sugarcane stalks is rich in longitudinally aligned fibrous tissues. These fibres are located farther from the neutral axis and thus possess a larger moment arm, enabling them to effectively resist the tensile and compressive strains generated during bending. As a result, the overall bending stiffness of the stalk is enhanced.

In this study, the equivalent bending response parameter is markedly higher than the equivalent radial deformation parameter, which may reflect a more pronounced contribution of fibrous tissues under bending conditions than under radial compression. This difference further suggests that the mechanical behaviour of sugarcane stalks may be associated with both tissue structural distribution and loading direction, although these anatomical factors were not directly quantified in the present study. The greater equivalent bending response of the basal section may be associated with longitudinal differences in tissue organisation and development. However, vascular bundle distribution, fibre content, lignification, and related anatomical characteristics were not directly measured in the present study. Therefore, this interpretation should be regarded as a possible mechanism rather than an experimentally demonstrated result.

By integrating the results of axial, radial, and bending mechanical property tests, it is observed that sugarcane stalks exhibit the highest stiffness in the axial direction, the lowest stiffness in the radial direction, and intermediate values for the bending response parameter, demonstrating a clear directional dependence. This mechanical behaviour is closely associated with the longitudinal alignment of vascular bundle fibres and the heterogeneous distribution of tissue structures across the cross-section, reflecting the typical transversely isotropic characteristics of sugarcane stalks.

#### 3.4.2 Correlation analysis between geometric dimensions and equivalent bending response parameter

To further investigate the influence of geometric dimensions on bending mechanical properties, linear regression relationships between specimen diameter and equivalent bending response parameter were established for both basal and middle-section specimens, respectively. The results are shown in **Figure 16**.

**Figure 16:**
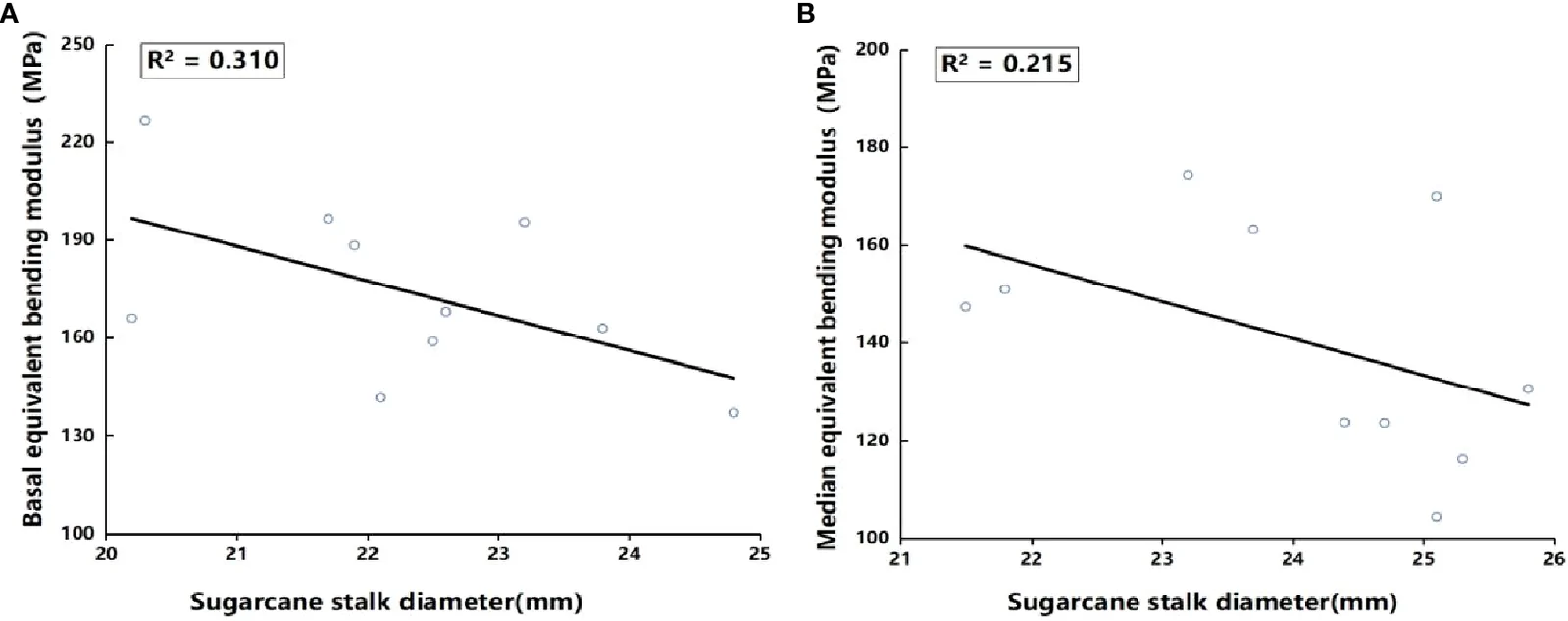
Coefficient of determination (*R²*) for the relationships between sugarcane stalk diameter and equivalent bending response parameter in the basal and middle sections (E_b_).

For basal specimens, the coefficient of determination R^2^ between diameter and equivalent bending response parameter is 0.31, while for middle-section specimens it is 0.215. Overall, both regression results remain at relatively low levels, indicating that only a weak linear correlation was observed between geometric dimensions and equivalent bending response parameter, and that geometric parameters have limited explanatory power for bending stiffness. These results suggest that both axial and radial compressive properties may be primarily associated with the intrinsic tissue structure of the material, with geometric dimensions playing a relatively minor role. In contrast, during three-point bending, the specimens are subjected to a combination of tensile, compressive, and shear stresses. As a result, the mechanical response is influenced not only by material properties but also by overall structural characteristics and cross-sectional effects. Therefore, compared with the axial elastic modulus (E_z_) and equivalent radial deformation parameter (E_r_), the equivalent bending response parameter (E_b_) exhibits higher sensitivity to variations in geometric dimensions, indicating that structural effects are more pronounced under bending conditions.

From a mechanical perspective, the three-point bending process involves a coupled response to tensile, compressive, and shear stresses. The mechanical behaviour may therefore be influenced not only by the constitutive properties of the material but also by the continuity of the internal fibrous structure, although this interpretation should be regarded as a possible mechanism rather than an experimentally demonstrated result. The weak correlations with diameter indicate that the measured geometric variable had limited explanatory power for the variation in E_b_. The observed basal–middle difference may also be associated with differences in internal tissue organisation, but these anatomical characteristics were not directly quantified in the present study.

### 3.5 Comprehensive comparative analysis of mechanical parameters of sugarcane stalks

To further elucidate the overall mechanical response characteristics of sugarcane stalks under different loading directions and at different internode positions, a comprehensive comparative analysis was conducted on the axial elastic modulus (E_z_), equivalent radial deformation parameter (E_r_), and equivalent bending response parameter (E_b_). The results are shown in **Figure 17**.

**Figure 17:**
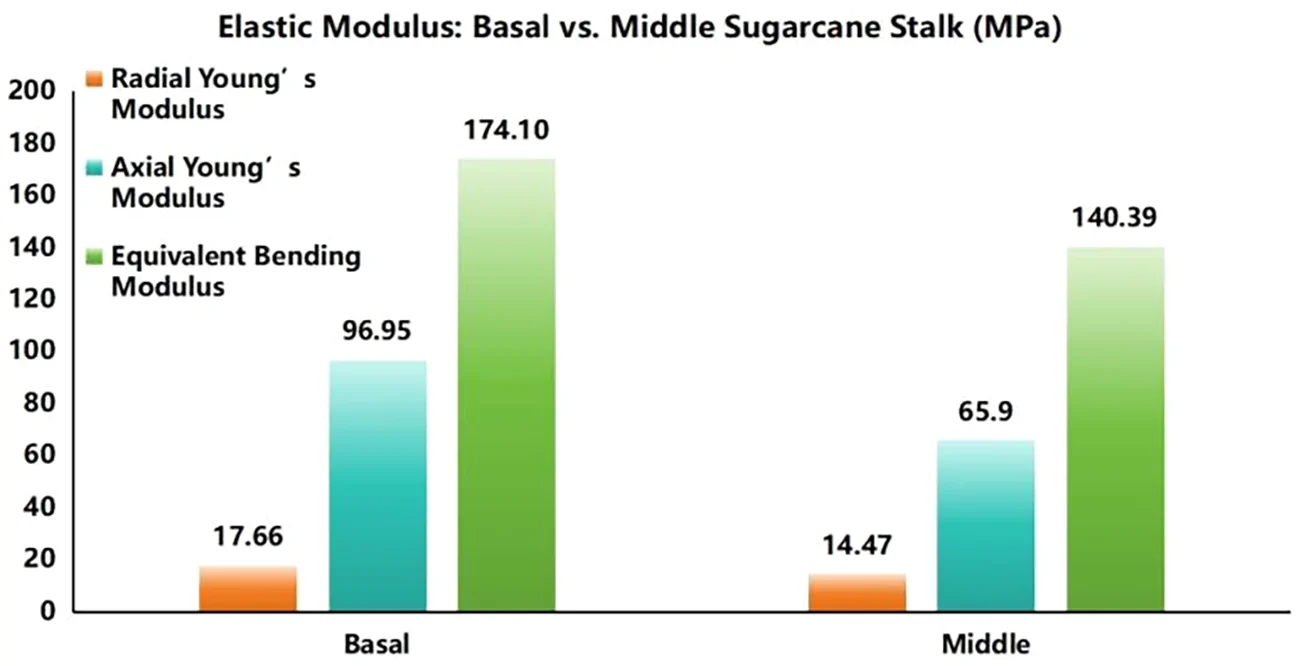
Comparison of axial elastic modulus (E_z_), equivalent radial deformation parameter (E_r_), and equivalent bending response parameter (E_b_) of sugarcane stalks between basal and middle sections.

The results show that the axial elastic modulus (E_z_), equivalent radial deformation parameter (E_r_), and equivalent bending response parameter (E_b_) of basal specimens are 96.95 MPa (95% CI: 85.07-108.83), 17.66 MPa (95% CI: 15.90-19.42), and 174.10 MPa (95% CI: 154.40-193.80), respectively, while those of middle-section specimens are 65.90 MPa (95% CI: 58.61-73.19), 14.47 MPa (95% CI: 12.58-16.35), and 140.39 MPa (95% CI: 123.13-157.64), respectively. Numerically, the measured parameters followed the relationship E_b_ > E_z_ > E_r_. All mechanical parameters of the basal section are significantly higher than those of the middle section (p < 0.05), indicating that sugarcane stalks exhibit both pronounced anisotropy and a longitudinal mechanical gradient. E_z_ is substantially higher than E_r_; specifically, the axial elastic modulus in the basal and middle sections is approximately 5.49 and 4.55 times greater than the equivalent radial deformation parameter, respectively (p < 0.001).Sugarcane stalks exhibit pronounced directional mechanical anisotropy and can be reasonably represented using a transversely isotropic modelling framework. Vascular bundles and fibrous tissues are continuously distributed along the longitudinal direction of the stalk, forming a natural fibre-reinforced architecture. Axial loads can therefore be efficiently transferred along the fibre direction, resulting in high axial stiffness. A similar fibre-oriented reinforcement mechanism is also widely observed in bamboo, wood, and other gramineous plants.

It is noteworthy that E_b_ is generally higher than E_z_. Since the equivalent bending response parameter (E_b_) is derived from the three-point bending response, it reflects the combined influence of material response, cross-sectional geometry, and the present bending configuration rather than an intrinsic elastic modulus. Therefore, the higher E_b_ primarily reflects the excellent overall bending resistance of sugarcane stalks rather than an increase in intrinsic material stiffness. The higher E_b_ value does not indicate that the intrinsic elastic modulus of sugarcane stalk tissues is higher than the axial modulus. Instead, it reflects the enhanced bending resistance caused by the interaction between the longitudinal fibre reinforcement and the geometric characteristics of the stalk cross-section. In addition to directional dependence, sugarcane stalks also exhibit a pronounced longitudinal mechanical gradient. Compared with the middle section, the basal section shows increases of 47.12%, 22.05%, and 24.01% in E_z_, E_r_, and E_b_, respectively. This longitudinal difference may be associated with variations in internal tissue organisation and developmental gradients along the stalk. The lower stiffness observed in the middle section may partly reflect differences in tissue development and mechanical demand, although anatomical characteristics such as vascular bundle distribution, fibre content, and lignification were not directly measured in this study.

Combined with the regression analysis results, it can be observed that the correlations between length and diameter and E_z_, E_r_, and E_b_ are relatively weak, with most coefficients of determination R^2^ below 0.30. This indicates that only a weak linear correlation was observed between geometric dimensions and mechanical properties, suggesting that external geometric dimensions are not the dominant factors governing the mechanical properties of sugarcane stalks.

The weak correlations with external geometric dimensions indicate that the measured length and diameter had limited explanatory power for the observed mechanical variation. However, these results alone do not identify the internal anatomical factors responsible for this variation. It should be noted that the equivalent bending response parameter (E_b_) is a structural response parameter obtained from three-point bending tests rather than an independent constitutive constant. Therefore, E_b_ was used to characterise the global bending behaviour of the stalk and to assess the agreement between the finite element predictions and the experimental bending response, whereas the transversely isotropic constitutive description was primarily established using E_z_ and E_r_. These findings provide an experimental basis for finite element modelling of sugarcane stalks. If a homogeneous isotropic material model is adopted, it is difficult to accurately represent their true mechanical behaviour. By contrast, introducing direction-dependent parameters such as E_z_ and E_r_ to construct a transversely isotropic model The results provide an experimental basis for developing a transversely isotropic FE representation of the macroscopic bending behaviour of sugarcane stalks under the tested conditions. In addition, the pronounced anisotropy and longitudinal gradient characteristics of sugarcane stalks suggest that mechanical differences across both loading directions and stalk positions should be carefully considered in mechanised harvesting processes. This is essential for optimising cutting, gripping, and conveying parameters, thereby improving operational performance and reducing mechanical damage.

### 3.6 Experimental comparison of the finite element model

#### 3.6.1 Quantitative comparison between simulation and experiment

The finite element results were compared with the three-point bending measurements for both basal and middle-section specimens. The results show that the average experimental peak displacement of the basal specimens is 6.86 mm, while the finite element prediction is 7.10 mm, corresponding to a relative error of 3.50%. For the middle-section specimens, the average experimental peak displacement is 7.14 mm, and the simulated value is 7.58 mm, yielding a relative error of 6.16%. In both cases, the peak displacement errors are below 10%, indicating that the model showed good agreement with the experimental peak-displacement response under the present three-point bending conditions, as shown in **Tables 12**, **13**.At the sample level, the displacement error of the basal specimens ranges from 2.5% to 8.4%, with an average error of 4.95%, while that of the middle-section specimens ranges from 3.7% to 7.7%, with an average error of 5.33%. In addition, the predicted peak loads from the finite element model are in good agreement with the experimental results, with all errors controlled within 10%.

**Table 12:** Three-point bending experimental–numerical comparison for basal specimens.

Group	Maximum load (N)	Peak displacement (mm)	Displacement relative error (%)	Load relative error (%)
Bending test group A	847.2	8.83	4.3	4.08
Bending simulation group A	812.6	9.23		
Bending test group B	683.4	5.11	4.1	3.99
Bending simulation group B	656.1	5.32		
Bending test group C	1316.0	8.70	2.5	2.39
Bending simulation group C	1284.5	8.48		
Bending test group D	818.0	5.44	5.1	4.78
Bending simulation group D	778.9	5.73		
Bending test group E	844.3	7.20	5.2	4.69
Bending simulation group E	804.7	7.58		
Bending test group F	806.0	5.70	8.4	7.59
Bending simulation group F	744.8	6.18		
Bending test group G	934.0	6.14	3.5	2.98
Bending simulation group G	906.2	6.36		
Bending test group H	1219.0	6.20	4.5	4.20
Bending simulation group H	1167.8	5.93		
Bending test group I	1106.5	6.10	4.4	3.92
Bending simulation group I	1063.1	6.37		
Bending test group J	1654.4	9.20	7.5	7.04
Bending simulation group J	1537.9	9.89		

**Table 13:** Three-point bending experimental–numerical comparison for middle-section specimens.

Group	Maximum load (N)	Peak displacement (mm)	Displacement relative error (%)	Load relative error (%)
Bending test group A	990.0	9.06	4.6	4.26
Bending simulation group A	947.8	8.70		
Bending test group B	994.0	8.40	5.5	4.94
Bending simulation group B	944.9	8.86		
Bending test group C	964.6	5.50	4.7	4.36
Bending simulation group C	922.5	5.76		
Bending test group D	1269.2	7.10	6.4	5.87
Bending simulation group D	1194.7	7.55		
Bending test group E	974.0	5.40	3.9	3.43
Bending simulation group E	940.6	5.19		
Bending test group F	1129.2	7.07	7.7	7.21
Bending simulation group F	1047.8	6.52		
Bending test group G	1309.1	9.10	3.7	3.38
Bending simulation group G	1264.8	9.44		
Bending test group H	1217.2	5.20	6.5	5.86
Bending simulation group H	1145.9	4.86		
Bending test group I	1355.7	8.20	4.3	3.99
Bending simulation group I	1301.6	7.85		
Bending test group J	1266.4	10.50	6.0	5.65
Bending simulation group J	1194.8	11.13		

Overall, the simulated peak displacement and peak load showed good agreement with the experimental measurements under the present quasi-static three-point bending conditions. These results support the ability of the model to reproduce the macroscopic pre-failure bending response under the conditions examined in this study.

#### 3.6.2 Consistency analysis of load–displacement curves

Based on the statistical results of 20 sets of parallel tests, the A-group basal specimens exhibit good apparent quality, with geometric parameters and mechanical properties closely matching the overall mean values. During the loading process, the force response is stable and the curves show no abnormal fluctuations, indicating excellent experimental stability. These specimens are therefore considered representative of the intrinsic mechanical behaviour of sugarcane in the corresponding section. Accordingly, the measured data from this group were selected for finite element simulation, and a load–displacement curve comparison for the basal sugarcane was conducted, as shown in **Figure 18**. The finite element results were compared with the three-point bending experiments to assess whether the model could reproduce the overall bending response under the present loading configuration. The bending data used for this comparison were obtained from the same experimental dataset reported in Section 3.4 and did not constitute an independent validation dataset. For comparison of the load–displacement trend, one representative experimental curve was selected from the bending dataset. The curve was selected according to its mechanical response being close to the average behaviour of the tested specimens rather than according to its agreement with the simulation.

**Figure 18:**
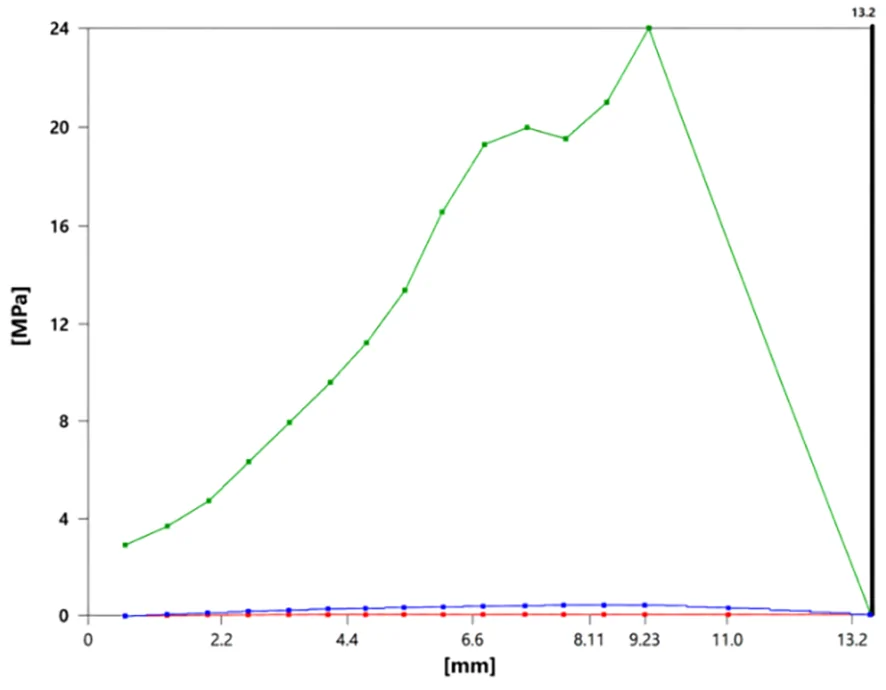
Simulated stress–displacement curve of basal sugarcane stalks.

The results show that the experimental and simulation curves exhibit a highly consistent overall trend. The simulation reproduced the nonlinear increase in load during the elastic deformation stage and showed good agreement with the overall deformation trend before failure. However, the experimentally observed rapid load decrease after the peak load was associated with stalk fracture, which was not captured by the current elastic model. Near the peak-load region, the experimental and simulated curves reached similar peak positions and exhibited consistent trends, indicating good agreement in the corresponding macroscopic response. The agreement of the load–displacement curves further indicates that the model can reasonably reproduce the overall pre-failure bending response under the present loading conditions.

#### 3.6.3 Analysis of deformation and stress distribution characteristics

**Figures 19**, **20** present the simulated displacement contour and Von Mises equivalent stress contour of a representative basal specimen under three-point bending conditions 6.

**Figure 19:**
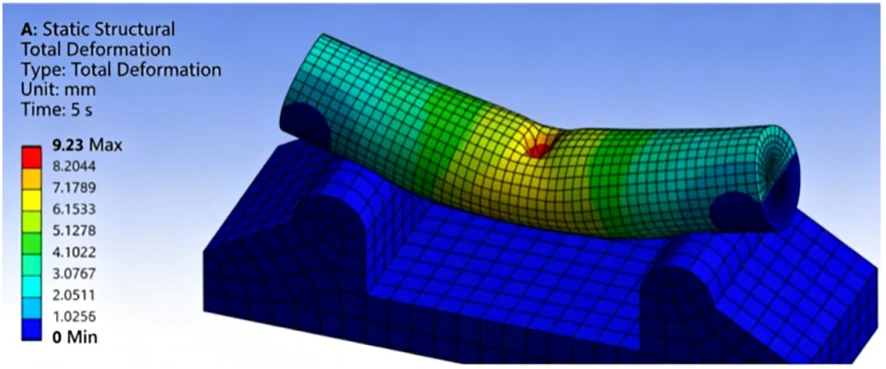
Displacement contour of basal specimen (simulation group A).

**Figure 20:**
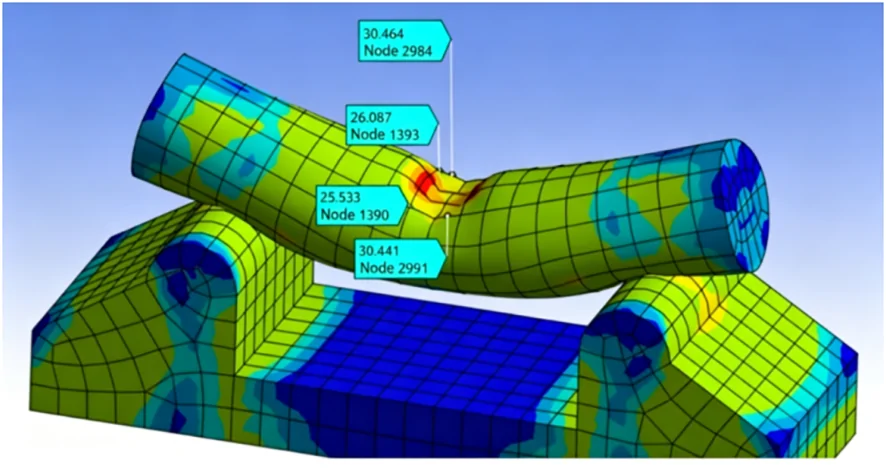
Von Mises equivalent stress contour of basal specimen (simulation group A).

From the displacement distribution results, it can be observed that the maximum displacement occurs at the mid-span loading region and gradually decreases towards the two supports along the span direction. The overall deformation pattern exhibits a typical simply supported beam bending behaviour, which is highly consistent with the experimentally observed bending morphology. This simulated deformation pattern was consistent with the experimentally observed macroscopic bending morphology under the present loading conditions.

To comprehensively characterize the multi-axial stress state inherent to the transversely isotropic material, the stress and strain tensors in the local material coordinate system (i.e., oriented along the stalk axis and the transverse plane) were extracted during post-processing. The longitudinal stress, transverse normal stresses in the isotropic plane, and shear components were extracted from the numerical model to examine the predicted directional stress state. These directional components provide a model-based interpretation of the possible internal load-transfer behaviour under the assumed transversely isotropic material representation and should not be regarded as experimentally measured internal stress fields.

From the stress distribution results, it can be observed that the maximum equivalent stress is mainly concentrated near the loading point and in the tensile region on the lower surface of the specimen. The upper surface is predominantly subjected to compressive stress, while the lower surface experiences tensile stress, and the stress level near the neutral axis is the lowest. This distribution is consistent with the classical beam bending theory. These results indicate that the model reproduces the global deformation response and predicts plausible internal stress-transfer paths and stress concentration regions under the assumed material and boundary conditions. However, because the internal stress and strain fields were not directly measured experimentally, these distributions should be interpreted as model-based predictions rather than experimentally validated observations. Considering the heterogeneous anatomical structure of sugarcane stalks, the local stress components in the material coordinate system would provide a more physically meaningful description of the anisotropic stress state. However, the present study focused on macroscopic deformation prediction, and insufficient experimental strength parameters were available to establish an anisotropic failure criterion.

In summary, comparison with the three-point bending experiments supports the ability of the finite element model to reproduce the macroscopic pre-failure load–displacement response. The internal stress and strain distributions obtained from the simulations provide additional model-based information on the predicted mechanical response, but these internal fields were not directly validated experimentally. Overall, the macroscale transversely isotropic finite element model, informed by the measured axial response and equivalent radial deformation response, showed good agreement with the experimental three-point bending response. The equivalent bending response parameter was used to characterise the global bending response and was not treated as an independent constitutive material constant. The comparison provides an experimental consistency check of the workflow linking mechanical parameter characterisation, finite element modelling, and three-point bending response. The agreement with representative three-point. These results provide a basis for further mechanical modelling under more representative harvesting-related loading conditions. However, the present experimental comparison should not be interpreted as validation of the model for loading conditions beyond quasi-static three-point bending or for damage and failure prediction.

Since bending loads are the most common and representative loading condition experienced by sugarcane stalks during mechanised harvesting, the three-point bending case was preferentially selected for finite element experimental comparison in this study. Although the present comparisons show good agreement between simulation and experiment under quasi-static three-point bending, they do not constitute validation against a completely independent dataset. Further work should evaluate the model using independently collected specimens and additional loading conditions.

## 4 Discussion

The present study shows that the mechanical behaviour of sugarcane stalks depends on both loading direction and longitudinal position. Axial resistance was consistently greater than radial resistance, while basal internodes exhibited higher stiffness than middle internodes. Previous studies on sugarcane and other crop stems have reported variations in mechanical behaviour with loading mode, stalk position, and structural characteristics (Oduntan et al., 2024; Shah et al., 2019; Stubbs et al., 2020; Bisht et al., 2022; Abdelhadi et al., 2023; Ma et al., 2024; Leblicq et al., 2015). However, these studies have generally examined individual mechanical responses or specific loading conditions separately. The present study integrates axial compression, radial compression, and bending within a common experimental framework, thereby allowing directional dependence and longitudinal variation to be characterised simultaneously and subsequently incorporated into a transversely isotropic finite element model.

The observed differences are consistent with the structural organisation of crop stems, in which longitudinally distributed vascular bundles and fibrous tissues contribute strongly to load transfer along the stalk axis (Fruehwald-Koenig and Heister, 2024; Speck and Burgert, 2011; Gangwar et al., 2021). The stronger basal response may similarly reflect differences in tissue development and mechanical demand along the stalk, although vascular bundle distribution, fibre content, lignification, and related anatomical characteristics were not measured directly in this study and therefore cannot be regarded as confirmed mechanisms. The weak relationships between the compression-derived parameters and external dimensions further indicate that stalk geometry alone cannot explain the observed mechanical variation. Bending requires a different interpretation because its response depends on both material stiffness and cross-sectional geometry. Previous analyses of plant stems have likewise shown that material properties and structural geometry jointly determine bending behaviour (Leblicq et al., 2015). Accordingly, E_b_ is referred to throughout this manuscript as an equivalent bending response parameter because it represents the combined material and structural response under the present three-point bending configuration rather than an independent constitutive elastic constant. Similarly, E_r_ is referred to as an equivalent radial deformation parameter because it represents the macroscopic radial deformation resistance obtained under the present diametral compression configuration rather than an intrinsic transverse Young’s modulus. In the homogenised finite element model, E_r_ was used as an effective transverse stiffness parameter at the macroscale.

Finite element models have been widely used to examine crop–machine interactions. In sugarcane, previous studies have focused mainly on task-specific processes such as chopping, cutting, and stalk damage to analyse failure mechanisms and optimise operating parameters (Xie et al., 2018; Qiu et al., 2021; Zhou et al., 2024). Related modelling studies in other crops have also shown that appropriate material representation is important for reproducing stem deformation during mechanical interaction (Leblicq et al., 2016; Li et al., 2023). The contribution of the present model therefore lies not in the use of finite element analysis itself, but in experimentally deriving direction-dependent equivalent stiffness parameters and incorporating them into a transversely isotropic representation of the sugarcane stalk. The agreement with the present three-point bending experiments indicates that this simplified formulation can reproduce the dominant macroscopic pre-failure response under the quasi-static three-point bending conditions examined here without assuming isotropic material behaviour. The model should nevertheless be regarded as a macroscale equivalent representation rather than a tissue-resolved description, and it is not intended to predict damage initiation, fracture, or post-failure behaviour.

From an engineering perspective, the directional and longitudinal dependence identified here indicates that both loading direction and stalk position should be considered when analysing clamping, feeding, conveying, and cutting processes. In particular, the lower transverse deformation resistance suggests that radial loading may be more sensitive to local deformation than loading transferred mainly along the stalk axis. These implications should, however, be interpreted within the experimental scope of this study. Only one mature cultivar, Guitang 21, was examined, and moisture content was not measured directly. Therefore, potential variation in specimen moisture and its contribution to the observed mechanical response could not be quantified in the present study. The anatomical basis of the observed mechanical differences was also not quantified. The Poisson’s ratios and density used in the finite element model were adopted from published values rather than measured directly for the specimens tested in this study. These assumed parameters may affect the predicted stiffness and load–displacement response and therefore represent a source of modelling uncertainty. Their influence was not quantified in the present study because a dedicated parameter sensitivity analysis was not performed. In addition, the bending parameter was derived using an engineering beam approximation under the present specimen geometry and loading configuration. Because the span-to-depth ratios were relatively small, transverse shear deformation, local contact deformation, and other non-ideal beam effects may have contributed to the measured displacement. These contributions could not be separated or quantified with the available experimental data and therefore represent a limitation of the present bending analysis. Its applicability to dynamic contact, cutting, impact, clamping, feeding, and progressive failure therefore remains to be established. Future work should combine multi-cultivar and controlled-moisture experiments with anatomical characterisation, improved bending formulations, multi-specimen experimental comparison, and dynamic or failure-related loading tests to extend the present framework towards actual harvesting conditions.

## 5 Conclusions

This study investigated sugarcane stalks of the cultivar ‘Guitang 21’. Axial compression, radial compression, and three-point bending tests were conducted to systematically obtain the axial elastic modulus (E_z_), equivalent radial deformation parameter (E_r_), and equivalent bending response parameter (E_b_) of basal and middle sections. The relationships between mechanical parameters and geometric dimensions were analysed, and a transversely isotropic finite element model was developed based on experimentally measured parameters. The main conclusions are as follows:

1. The mature Guitang 21 sugarcane stalks tested under the present experimental conditions exhibited pronounced directional dependence in their macroscopic mechanical response. The axial response was substantially greater than the equivalent radial deformation response. The equivalent bending response parameter showed the largest numerical value among the three reported parameters. However, because E_b_, E_r_, and E_z_ do not represent three equivalent intrinsic material constants, their numerical values should not be interpreted as a direct comparison among intrinsic elastic moduli. The results suggest that sugarcane stalks can be essentially regarded as natural fibre-reinforced biological composites, whose load-bearing behaviour is strongly influenced by the directionally distributed vascular bundles and fibrous tissues, as suggested by the measured directional dependence of the mechanical response (Speck and Burgert, 2011). Compared with the radial direction, the axial direction provides higher load transfer efficiency and deformation resistance; therefore, the assumption of a homogeneous isotropic material is insufficient to accurately represent their true mechanical behaviour.
2. The tested mature Guitang 21 stalks exhibited a clear longitudinal difference in mechanical response between the basal and middle sections under the present experimental conditions. The basal region presents significantly higher values of E_z_, E_r,_ and E_b_ than the middle section, with increases of approximately 47.12% in axial elastic modulus and 24.01% in equivalent bending response parameter, and the differences are statistically significant (p < 0.05). These differences may be associated with longitudinal variations in internal tissue organisation; however, the relevant anatomical variables were not directly quantified in this study.
3. Different mechanical parameters exhibit significantly different sensitivity to geometric dimensions. E_z_ and E_r_ showed generally weak linear correlations with specimen length and diameter, indicating that the tested geometric variables had limited explanatory power for the corresponding compressive responses. However, Er remains an equivalent radial deformation parameter defined by the present diametral compression configuration and should not be interpreted as an intrinsic transverse elastic constant. In contrast, E_b_ reflects the combined influence of material response and structural geometry under three-point bending and shows a certain degree of correlation with diameter, suggesting that bending response is jointly influenced by both material properties and structural geometry. These results suggest that, for the tested Guitang 21 specimens, the bending response under the present three-point bending configuration was influenced jointly by material response and structural geometry. In bending-dominated conditions, geometric factors should not be neglected, and different mechanical parameters should be treated distinctly in modelling and engineering analyses.
4. The present macroscale transversely isotropic finite element model showed good agreement with the measured macroscopic response of the tested Guitang 21 specimens under the present quasi-static three-point bending conditions. The predicted peak load and load–displacement response were consistent with the experimental measurements, with peak-load errors below 10%. These results support the ability of the model to reproduce the global pre-failure bending response under the specific conditions examined in this study. This experimental comparison does not establish general predictive validity for other loading conditions, dynamic processes, or damage and failure behaviour.
5. This study provides a mechanical characterisation and modelling framework for mature Guitang 21 sugarcane stalks under the specific quasi-static loading conditions investigated. The measured directional and longitudinal differences may provide useful reference information for future mechanical analyses related to sugarcane harvesting. However, the present model has not been evaluated for dynamic contact, cutting, impact, clamping, feeding, conveying, or progressive failure processes. Further experimental and modelling studies under these conditions, as well as studies involving other cultivars, maturity stages, and environmental conditions, are required before the present findings can be generalised or applied to specific harvesting component design.

## Data Availability

The original contributions presented in the study are included in the article/supplementary material. Further inquiries can be directed to the corresponding authors.
